# Synthetic Receptors Based on Abiotic Cyclo(pseudo)peptides

**DOI:** 10.3390/molecules27092821

**Published:** 2022-04-28

**Authors:** Stefan Kubik

**Affiliations:** Fachbereich Chemie—Organische Chemie, Technische Universität Kaiserslautern, Erwin-Schrödinger-Str. 54, 67663 Kaiserslautern, Germany; kubik@chemie.uni-kl.de

**Keywords:** supramolecular chemistry, molecular recognition, synthetic receptors, cyclopeptides, cyclic pseudopeptides, amino acids

## Abstract

Work on the use of cyclic peptides or pseudopeptides as synthetic receptors started even before the field of supramolecular chemistry was firmly established. Research initially focused on the development of synthetic ionophores and involved the use of macrocycles with a repeating sequence of subunits along the ring to facilitate the correlation between structure, conformation, and binding properties. Later, nonnatural amino acids as building blocks were also considered. With growing research in this area, cyclopeptides and related macrocycles developed into an important and structurally diverse receptor family. This review provides an overview of these developments, starting from the early years. The presented systems are classified according to characteristic structural elements present along the ring. Wherever possible, structural aspects are correlated with binding properties to illustrate how natural or nonnatural amino acids affect binding properties.

## 1. Introduction

In a brief personal account entitled “Why Cyclic Peptides? Complementary Approaches to Conformations”, Deber, Madison, and Blout wrote in 1976: “In choosing molecules which might be suitable models for conformational analysis, our goal has been to combine biological relevance with structural simplicity […]” [1]. The review then focuses on the structural characterization of small synthetic cyclopeptides, but the Blout group also used the guidelines derived from these analyses to identify macrocycles able to interact with suitable binding partners such as metal ions or amino acids [2]. With this early work, Blout contributed to a branch of supramolecular chemistry, a research field that was still in its infancy in the 1970s [3], that is devoted to the development of synthetic cyclopeptide-derived receptors [4,5,6,7,8,9]. Many of the receptors later described by other groups follow the original principle of combining structural simplicity with the ability of the amino acid-derived subunits to exert conformational control. Besides the relatively predictable binding properties of such hosts, additional advantages are their chirality, which can give rise to enantioselective substrate recognition, and the possibility to produce them via sequential syntheses, allowing a relatively facile exchange of individual building blocks. Moreover, the close structural relationship between cyclopeptides and proteins permits investigating and imitating fundamental principles of molecular recognition processes relevant in Nature.

This review provides an overview of work in this area, beginning with the early studies by Blout and others and then up to more recent developments. The presented receptors are classified according to structural aspects of the individual subunits, the subunit sequence along the ring, and the presence of linking units other than peptide bonds. In this context, the term cyclopeptide is restricted to macrocycles with a continuous unidirectional sequence of amino acids along the ring, including compounds in which some or even all subunits are nonnatural. All other types of receptors are termed pseudopeptides. Work is particularly highlighted in which a structural characterization of the receptor is combined with the evaluation of binding properties. Only macrocyclic receptors are considered, although acyclic [6,8] or cage-type amino acid-derived receptors [10] also exist. In addition, self-assembling cyclic peptides and pseudopeptides are not discussed, although their behavior is also due to the conformational control of the subunits. The resulting conformations are self-complementary, however, and not preorganized to recognize an ion or molecule. Individual rings thus stack into tubular arrangements in which the interactions between rings resemble those in the parallel or antiparallel β-sheets of proteins. Readers interested in these macrocycles are referred to relevant reviews [11,12,13].

## 2. Cyclic Peptides and Pseudopeptides Containing Only α-Amino Acid-Derived Subunits

**Scheme 1:**
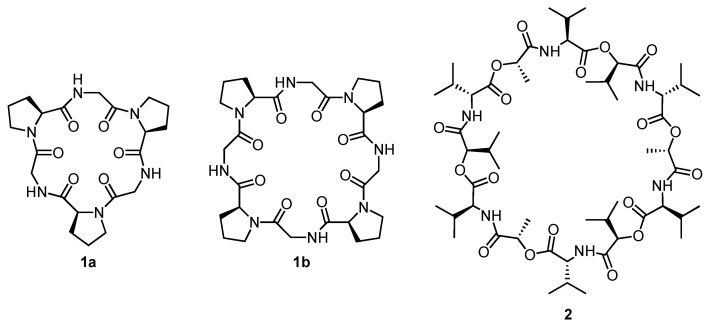
Structures of cyclopeptides **1a**,**b** and of valinomycin **2**.

The work of the Blout group on the solution structures of cyclic peptides initially focused on *cyclo*(Pro-Gly)_3_ **1a** and *cyclo*(Pro-Gly)_4_ **1b** (Scheme 1).

**Figure 1:**
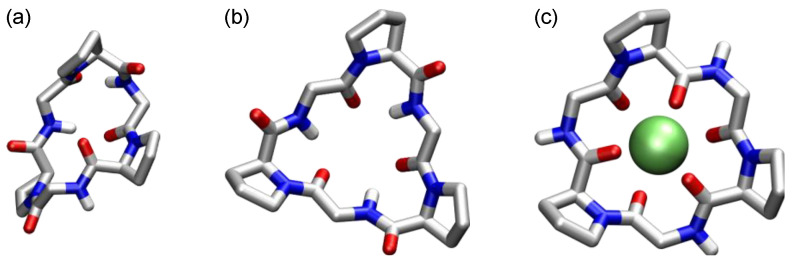
Calculated structures of the preferred conformation of **1a** in polar solvents (**a**), in nonpolar solvents (**b**), and structure of the 1:1 complex with Ca^2+^ (**c**). The calculations were performed by the author of this review based on the reported results by using Spartan 20 (Wavefunction, Inc., Irvine, CA, USA) and the MMFF force-field (see Supporting Information). Protons, except acidic ones, are omitted for reasons of clarity. For the proposed 2:1 structure of the Mg^2+^ complex, no *C*_3_ symmetric structure could be obtained.

NMR spectroscopic studies combined with circular dichroism and computational methods indicated that **1a** adopts an asymmetric conformation in water or DMSO with a *cis*-amide bond at one proline unit [14]. In less polar solvents such as 1,4-dioxane and chloroform, **1a** is *C*_3_ symmetric and stabilized by three intramolecular hydrogen bonds. The corresponding so-called *S* conformation undergoes a conformational reorganization in the presence of monovalent or divalent metal ions to allow for cation coordination. The interaction with Ca^2+^ induces the *S*_1_* conformation in which all six carbonyl oxygen atoms converge, allowing them to interact with the cation. In the Mg^2+^ complex of **1a**, two metal ions are bound to a related *S*_2_* conformation. To illustrate these arrangements, calculated structures of **1a** and its 1:1 Ca^2+^ complex are depicted in Figure 1.

**Figure 2:**
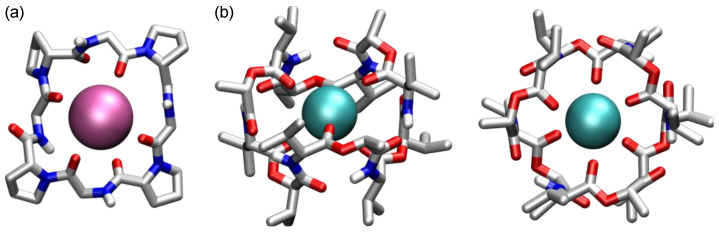
Crystal structure of the Rb^+^ complex of **1b** (CSD Entry: CPRGLR) (**a**), and side and top view of the crystal structure of the K^+^ complex of **2** (CSD Entry: TEFBAH) (**b**). Solvent molecules, counterions, and protons, except acidic ones, are omitted for reasons of clarity.

The larger cyclopeptide **1b** behaves analogously, adopting asymmetric conformations with *cis*-amides in polar solvents, an all-*trans* symmetric conformation in apolar solvents, and a second all-*trans* conformation when interacting with metal ions [2]. The latter conformation resembles the one found in the crystal structure of the Rb^+^ complex of **1b**, in which the four glycine carbonyl groups coordinate to the metal ion [15]. Three proline carbonyl groups also converge toward the center of the ring, while the fourth group points away, likely because it interacts with a water molecule in the crystal (Figure 2a).

The work of the Blout group not only contributed to the understanding of structural aspects of cyclopeptides, but also provided insight into their receptor properties. For example, both peptides **1a** and **1b** were shown to bind alkaline earth metal ions. The hexapeptide prefers Ca^2+^, with the corresponding complex having a *K*_a_ of 1400 M^−1^ in 80 vol% methanol/water, while the stability of the Ba^2+^ complex is lower by a factor of ca. 3 [14]. This selectivity reverses for **1b**, likely because of the larger diameter of this macrocycle [2]. In organic solvents, both peptides also form higher complexes with metal ions and interact with the ammonium salts of amino acid esters. The hydrochloride of L-Pro-Bn forms a discrete 1:1 complex with **1a** in chloroform, for example, while up to two molecules of L-Val-OMe·HCl are bound under the same conditions [16]. Complex formation is due to hydrogen bonds between the NH groups of the guests and the cyclopeptide carbonyl groups. When using racemic amino acids as substrates, the corresponding diastereomeric complexes are distinguishable by NMR spectroscopy [17].

This work, most of which was performed in the 1970s, was clearly inspired by the discovery of crown ethers at the end of the 1960s [18] and the almost simultaneous elucidation of the mode of action of antibiotics such as valinomycin (**2**, Scheme 1), which act as ionophores and transport metal ions across lipid membranes [19]. Abiotic but bioinspired cyclopeptides were expected to give rise to compounds with similar bioactivities. To illustrate the binding mode of valinomycin, the structure of the potassium complex is depicted in Figure 2b [20]. In this complex, valinomycin adopts a bracelet-like structure, which is stabilized by hydrogen bonds between NH groups and valine CO groups. The resulting converging arrangement of the ester carbonyl groups is ideally suited for K^+^ coordination. While Rb^+^ can also be bound, the cavity is too large to efficiently bind smaller ions such as Na^+^ or even Li^+^. The resulting selectivity is impressive: the K^+^ complex of **2** has a log *K*_a_ of 4.5 in methanol, whereas the stability of the Na^+^ complex is more than three orders of magnitude smaller (log *K*_a_ = 0.8) [21]. Important contributions to the development of valinomycin mimics not only came from the Blout group [22], but also from the groups of Ovchinnikov [23] and Gisin [24]. Although efficient metal binders were identified, especially on the basis of cyclopeptides structurally more complex than **1a** and **1b**, no cyclopeptide that could rival **2** in terms of binding properties and bioactivity emerged. The Ca^2+^-binding cyclic decapeptide *cyclo*[Pro-Phe-Phe-Ala-Glu(O*t*Bu)]_2_ is a recent addition to this family [25]. For an overview of metal-binding peptides, see [26].

**Scheme 2:**
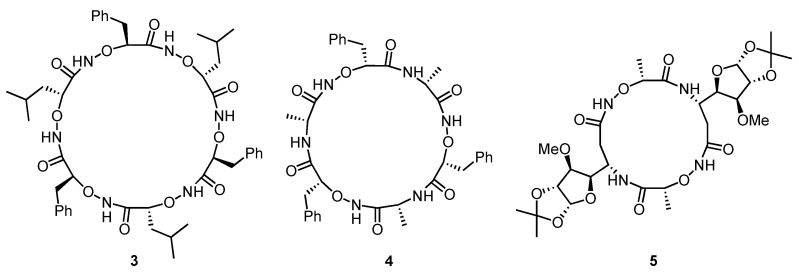
Structures of the cyclic pseudopeptides **3**, **4**, and **5** containing α-aminoxy acid subunits.

**Figure 3:**
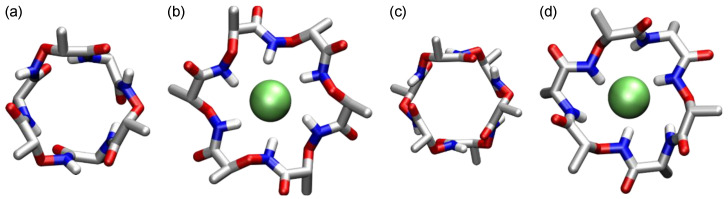
Calculated structures of uncomplexed **3** (**a**) and its chloride complex (**b**), and of uncomplexed **4** (**c**) and its chloride complex (**d**). The calculations were performed by the author of this review based on the reported results by using Spartan 20 (Wavefunction, Inc., Irvine, CA, USA) and the MMFF force-field (see Supporting Information). The side chains of both cyclic pseudopeptides are truncated and protons, except acidic ones, are omitted for reasons of clarity.

Bracelet-like conformations, somewhat related to the conformation of **2** in its K^+^ complex, can also be found in cyclic oligomers of α-aminoxy acids [27,28,29]. α-Aminoxy acids are derivatives of β-amino acids in which the β-carbon atom is replaced by an oxygen atom. Alternatively, they can be regarded as derivatives of α-amino acids containing an additional oxygen atom between the α-carbon and the amino group. Oligomers of these compounds are conformationally stabilized by hydrogen bonds between carbonyl groups and NH groups in neighboring subunits, giving rise to cyclic eight-membered arrangements: so-called α-N−O turns [27]. Chains containing homochiral α-aminoxy acids fold into helical structures, while those containing alternating *R*- and *S*-configured subunits form loops. The latter systems are predisposed to cyclize, leading to rigid macrocycles with all NH and CO groups involved in intramolecular hydrogen bonds. To illustrate this arrangement, the calculated structure of the cyclic hexamer **3** (Scheme 2) is depicted in Figure 3. The addition of a chloride salt to a solution of **3** in dichloromethane induces a conformational reorganization, leading to a structure in which a converging arrangement of the NH groups allows them to simultaneously interact with the anion [27]. In contrast to the ionophores discussed earlier, these pseudopeptides therefore act as anion receptors [8]. The log *K*_a_ of the chloride complex of **3** amounts to 4.1 in CD_2_Cl_2_. The cyclic hexamer **4** (Scheme 2), comprising alternating (*R*)-α-aminoxy acids and (*R*)-α-amino acids, behaves in a similar manner, forming a complex that is slightly more stable than that of **3** (log *K*_a_ = 4.2) (Figure 3) [30]. Other tested anions (bromide, iodide, nitrate) are bound at least 16 times less strongly. The pronounced anion affinity also enables **4** to extract chloride salts from water to chloroform. The even smaller cyclic pseudopeptide **5** described by the Kunwar group binds halides with a binding mode that is similar to that of **3** [31]. The respective *K*_a_ values of the chloride and bromide complexes only amount to 513 M^−1^ and 111 M^−1^ in CDCl_3_, however, which was attributed to the small diameter of **5**. Iodide binding turned out to be too weak for quantification.

A peptide-inspired structural motif that has found widespread application in the design of foldamers and bioactive molecules is that of peptoids [32]. Peptoids are glycine oligomers with *N*-substituted peptide bonds. They can formally be regarded as peptides in which the amino acid side chains are shifted by one position from the α-carbon to the nitrogen atom in each subunit. Since the side chains can be varied in a wide range, peptoids are structurally more diverse than peptides comprising only natural amino acids. In addition, the conformational behavior of peptoids is often relatively complex because *cis*-amides and *trans*-amides can exist at every subunit, while *cis*-amides in peptides usually only occur at proline residues.

**Figure 4:**
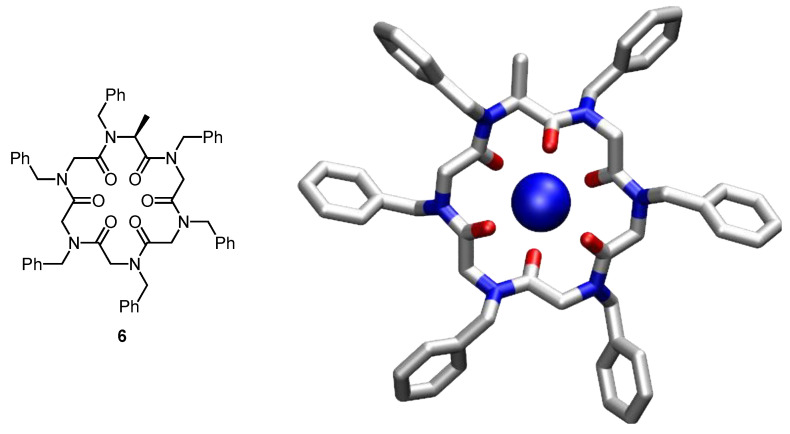
Structure of the cyclic peptoid **6** and calculated structure of its Na^+^ complex. The calculations were performed by the author of this review based on the reported results by using Spartan 20 (Wavefunction, Inc., Irvine, CA, USA) and the MMFF force-field (see Supporting Information). Protons are omitted for reasons of clarity.

Cyclic peptoids were first introduced in 2007 [33], and a major focus of the subsequent work in this area has been the development of bioactive derivatives [34]. In this context, structural studies played an important role to gain insight into how parameters such as ring size or the nature of the substituents influence the conformational behavior [35,36]. In some cases, the effects of metal or ammonium ions on the conformation were also studied, that is, of substrates that also interact with cyclic peptides or pseudopeptides. The cyclic peptoid **6** (Figure 4) adopts multiple conformations in chloroform, for example, that slowly interconvert on the NMR time scale and that likely differ in the number and relative positions of *cis*-amides along the ring [37]. In the presence of Na^+^, however, **6** adopts a symmetric all-*trans* conformation with the six carbonyl groups converging and coordinating to the metal ion (Figure 4). Similar effects have been observed for other cyclic peptoids, although in the case of larger rings, the formation of higher complexes containing more than one metal ion (or another cationic guest) was also observed [38,39,40,41].

There are also a number of receptors in which cyclopeptide ring mainly serve as scaffolds with their side chains mediating substrate binding [42,43,44,45,46,47,48]. Since the cyclic part of the receptor only ensures the proper orientation of the substituents without being immediately involved in substrate binding, these systems are not described here.

## 3. Cyclic Pseudopeptides Containing Cystine Subunits

**Scheme 3:**
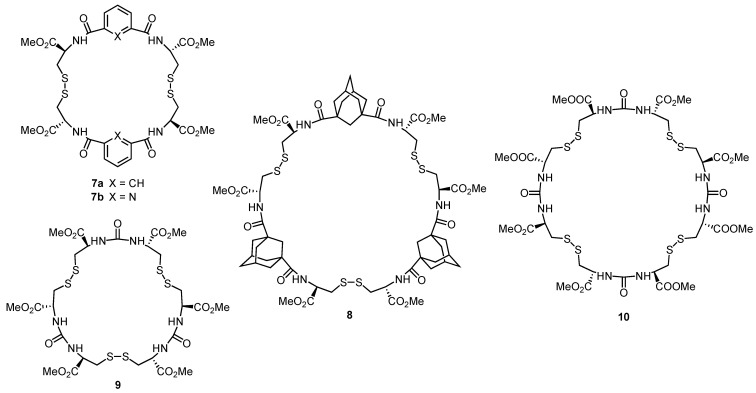
Structures of the cystine-containing macrocyclic pseudopeptides **7**–**10**.

Examples of macrocyclic receptors containing one or more cystine subunits are depicted in Scheme 3. These compounds can conveniently be obtained by treating a cystine diester with a suitable dicarboxylic acid chloride under basic conditions. In this way, different macrocycles are usually produced simultaneously, which can be subsequently separated chromatographically and/or by crystallization [49].

**Figure 5:**
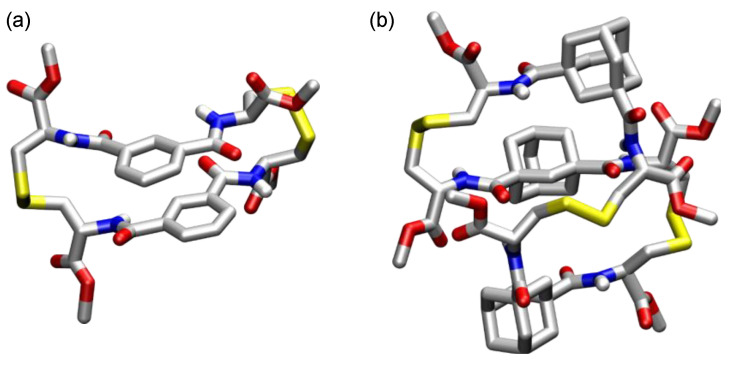
Crystal structures of **7a** (CSD Entry: NOSQIV) (**a**) and **8** (CSD Entry: TUHRAP) (**b**). Solvent molecules and protons, except acidic ones, are omitted for reasons of clarity.

In terms of structure, the incorporation of S–S bonds into a ring has several consequences [50]. First, disulfide groups are relatively rigid (the rotational barrier of an S–S bond is intermediate between those of C–C single bonds and amide bonds) with a preferred absolute dihedral angle of 90°. Disulfide bonds thus induce folding, as demonstrated by the solid-state structures of **7a**, in which the two S–S bonds produce an almost parallel arrangement of the two aromatic units [49], and **8**, which has an unusual figure of eight conformation (Figure 5) [51]. In addition, cystine units invert the directionality of the amide bonds. Accordingly, the compounds shown in Scheme 3 do not represent cyclopeptides in the strict sense (although they are sometimes termed cyclopeptides in the literature) but should better be termed pseudopeptides.

Major contributions to the development and structural characterization of cystine-containing cyclic pseudopeptides, of which compounds with aromatic subunits were termed cystinophanes [49], came from Karle, Ranganathan, and coworkers [52]. This work particularly focused on the self-assembling properties of such macrocycles, but several studies also addressed receptor properties. In this context, the cystinophane **7b** was shown to bind dicarboxylic acids in chloroform [49]. The *K*_a_ of the glutarate complex in CDCl_3_ amounts to 370 M^−1^, for example, and complex formation was attributed to the formation of hydrogen bonds between the carboxylate oxygen atoms and the peptide NH groups. The cystine-based cyclic oligourea derivatives **9** and **10** were shown to bind anions with moderate affinity [53]. For example, the stability constants of the chloride complexes of **9** and **10** in CDCl_3_ amount to, respectively, 2050 M^−1^ and 200 M^−1^. The Chen group also described a number of cyclic pseudopeptides with cystine units [54,55,56,57]. These compounds were shown to bind cations and/or anions in organic media, but no detailed structural information is available that allows correlating the binding properties with structural parameters.

**Scheme 4:**
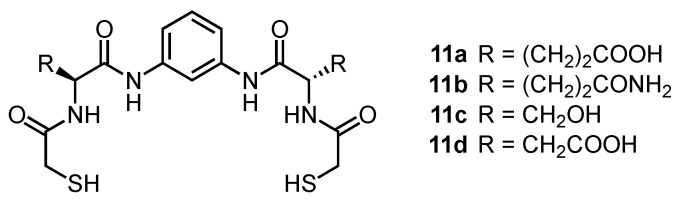
Structures of the dithiols **11a**–**d** used to generate macrocycles by oxidative disulfide formation and disulfide exchange.

Another approach to synthesize disulfide-containing cyclopeptides is to couple terminal thiol groups of suitable building blocks under oxidative condition. Reversible disulfide exchange at these linkages then allows the outcome of the reaction to be controlled by the presence of a suitable template. This dynamic combinatorial chemistry [58] strategy was used by Alfonso and coworkers to control the macrocyclization of the amino acid-derived building blocks **11a**–**d** (Scheme 4) [9,59]. They showed in this context that building blocks **11a**–**c** produce a mixture of macrocycles of different ring sizes with the macrocyclic dimers dominating. The addition of salts to this mixture causes the amplification of the homodimer containing two glutamic acid-derived subunits **11a**, indicating that macrocycles with polar acidic side chains are thermodynamically favored at high ionic strengths. This effect is even more pronounced for the aspartic acid-derived building block **11d**.

## 4. Cyclic Pseudopeptides Containing Nonnatural Aliphatic Subunits

**Figure 6:**
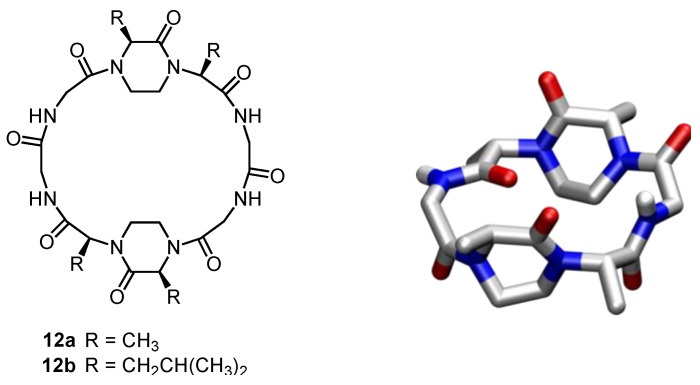
Structures of the cyclic pseudopeptides **12a**,**b** and crystal structure of **12a** (CSD Entry: ZARZOH). Solvent molecules and protons, except acidic ones, are omitted for reasons of clarity.

Miyake and Kojima investigated the structure and receptor properties of macrocyclic pseudopeptides containing *N*,*N*′-ethylene-bridged dipeptide units [60]. An example is compound **12a** (Figure 6), which formally derives from a cyclic octapeptide, in which the nitrogen atoms of the first and second and those of the fifth and sixth amino acid residues are linked with ethylene units. Alternatively, this macrocycle can be regarded as a cyclic pseudopeptide containing two piperazin-2-one units. These units prevent the formation of intramolecular hydrogen bonds and therefore have a pronounced effect on the folding pattern. They also reduce the conformational mobility because they prevent rotation around certain N–C(α) and C(α)–CO bonds, but the presence of the tertiary amides at the same time increases the probability of *cis*-amides along the ring. While NMR spectroscopy indicated that *trans*-amides prevail in solution, one of the six amide bonds of **12a** has a *cis*-conformation in the solid state [61]. Binding studies showed that **12b** binds divalent alkaline earth and transition metal ions [62,63]. In addition, the formation of diastereotopic complexes was observed for some of these pseudopeptides by ^13^C NMR spectroscopy in the presence of racemic ammonium ions [64].

Cyclic pseudopeptides with alternating natural α-amino acid and oxazolidin-2-one residues have also been described [65], and although acyclic pseudopeptides containing these building blocks have been shown to assemble into fibers and gels [66], little is known about the binding properties of the cyclic counterparts. Ranganathan and Karle prepared cyclic pseudopeptides containing adamantane residues or used the OH group in the side chains of serine or tyrosine to prepare cyclic depsipeptides [52]. These compounds were, however, mainly studied with regard to their membrane transport and self-assembly properties.

## 5. Cyclic Peptides and Pseudopeptides Containing Six-Membered Aromatic Subunits

The introduction of 2-, 3- or 4-aminobenzoic acid derivatives into cyclopeptides leads to hybrid cyclophane-cyclopeptide receptors in which the aromatic subunits serve to reduce conformational flexibility, enlarge the ring, and inhibit the formation of intramolecular hydrogen bonds that could cause a collapse of the cavity. The aromatic subunits also allow the introduction of further substituents, and their π-surfaces can mediate interactions with electronically complementary binding partners.

**Figure 7:**
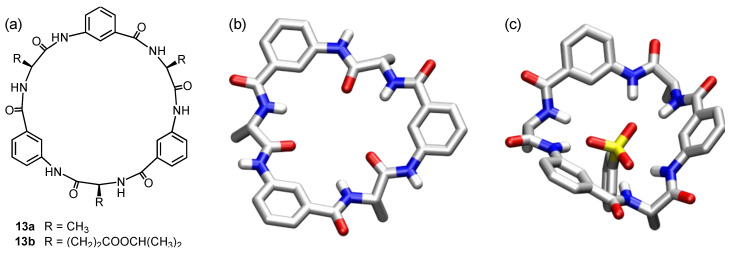
Structures of the cyclic peptides **13a**,**b** (**a**), crystal structure of **13a** (CSD Entry: XEHMUW) (**b**), and calculated structure of the benzenesulfonate complex of **13a** (**c**). The calculations were performed by the author of this review based on the reported results by using Spartan 20 Wavefunction, Inc., Irvine, CA, USA) and the MMFF force-field (see Supporting Information). Solvent molecules, counterions, and protons, except acidic ones, are omitted for reasons of clarity.

Pioneering work in this area was conducted by Ishida and coworkers, who showed that cyclopeptides that contain an alternating sequence of aliphatic α-amino and 3-aminobenzoic acids, thus representing formal analogs of metacyclophanes, interact with anions in DMSO [67]. The cyclic hexapeptide **13a** (Figure 7a), for example, forms a 1:1 complex with 4-nitrophenyl phosphate with a remarkable log *K*_a_ of 6.1. Exchanging the alanine units for other α-amino acids does not have a strong effect on anion binding, but the corresponding octapeptide has a circa two orders of magnitude lower phosphate affinity. Anion complexation was attributed to hydrogen bond formation between the NH groups of **13a** and the oxygen atoms of the anion, and since both the aromatic and aliphatic NH protons are deshielded in the ^1^H NMR spectrum upon complex formation, the cyclopeptide was believed to adopt a conformation in the complex in which all six NH groups converge. Although this assumption was later confirmed by Kubik et al. [68], a crystal structure revealed that **13a** prefers a different conformation in the solid state in which only the aliphatic NH groups converge, while the aromatic NH groups point away from the cavity center (Figure 7b) [69]. All amide groups have *trans*-conformations and the side-chain methyl groups are oriented on the same side of the ring. Ishida subsequently demonstrated that such cyclopeptides can also serve as mimics for serine protases [70] and to mediate membrane transport [71].

The receptor properties of the better soluble cyclohexapeptide **13b** that contains glutamic acid-derived subunits were studied in 0.2 vol% DMSO-*d*_6_/CDCl_3_ by Kubik [68]. These investigations showed that **13b** has fewer degrees of conformational freedom in the less polar solvent mixture than in DMSO. According to NOESY NMR spectroscopy, a conformation dominates with the aromatic NH groups oriented toward H(4) of the neighboring aromatic subunit, while the glutamic acid NH groups point toward H(2). Thus, the preferred conformation of **13b** in the chloroform mixture likely resembles that of **13a** in the solid state (Figure 7b).

Since the aromatic subunits in **13a** and **13b** increase the distance of the carbonyl groups with respect to cyclic hexapeptides containing only α-amino acids (e.g., **1a**), a complexation of metal ions or protonated ammonium groups is no longer possible. Therefore, the complexation of quaternary ammonium ions that are known to bind to cyclophanes by cation-π interactions was studied [72,73]. Small upfield shifts of the cation signals were indeed observed in the ^1^H NMR spectrum when **13b** was added to a solution of *n*-butyltrimethylammonium (BTMA) iodide in 0.2 vol% DMSO-*d*_6_/CDCl_3_, accounting for an interaction. However, with a *K*_a_ of 300 M^−1^, the stability of the complex formed in this way is only moderate.

In comparison to BTMA iodide, the respective tosylate salt causes much more pronounced changes in the ^1^H NMR spectrum. Not only are the complexation-induced upfield shifts of the cation signals much stronger, the rather broad cyclopeptide signals also sharpen considerably. In addition, the exchange of iodide for tosylate causes an increase of the apparent stability of the BTMA complex by a factor of ca. 10^4^. These effects were attributed to the binding of the tosylate anion to the cyclopeptide in an arrangement calculated for the benzenesulfonate complex of **13a** on the basis of NMR spectroscopic evidence (Figure 7c). The result is consistent with the structure of the phosphate complex of **13a** proposed by Ishida [67]. Tosylate binding thus rigidifies the cyclopeptide and preorganizes it for cation binding. However, the main reason for the high stability of the cation complex mainly is the Coulomb attraction of the two simultaneously bound ions. The tosylate anion thus acts as an allosteric effector in that it strengthens the binding of the substrate (the cation) to the receptor (the cyclopeptide) by binding to an independent binding site and causing a conformational reorganization.

**Figure 8:**
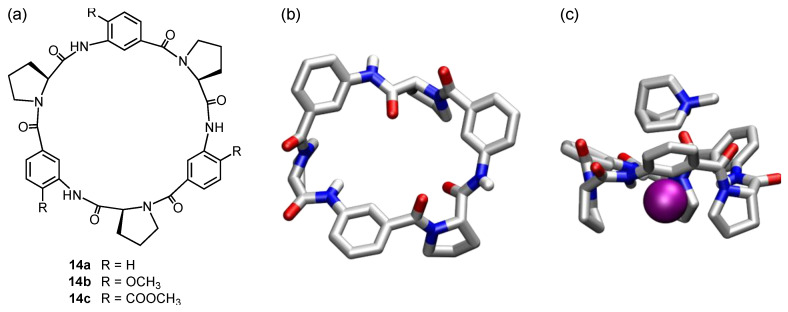
Structures of the cyclic peptides **14a**–**c** (**a**), crystal structure of **14a** (CSD Entry: KIQXUD) (**b**), and crystal structure of the *N*-methylquinuclidinium iodide complex of **14a** (CSD Entry: KIQYAK) (**c**). Solvent molecules, counterions, and protons, except acidic ones, are omitted for reasons of clarity.

A similar behavior was observed for the structurally related proline-containing cyclopeptide **14a** (Figure 8a) [74]. This cyclopeptide adopts a nonsymmetric all-*trans* conformation in the solid state, differing in the arrangements of the residues between each pair of aromatic subunits (Figure 8b). While all three aromatic carbonyl groups have similar orientations, pointing more or less into the direction of the aromatic H(6) protons, the arrangements of the three secondary amides differ more strongly. One of these groups orients the NH proton toward H(2) of the neighboring aromatic ring, while the NH protons of the other two amide groups point toward H(4). No intramolecular hydrogen bonds exist between NH and CO groups as in **1a** (Figure 1a), showing that the replacement of the glycine units in **1a** by 3-aminobenzoic acid subunits has more profound structural effects than just increasing the diameter of the ring.

While calculations indicate that the nonsymmetric conformation in the crystal could indeed be the thermodynamically most stable one, **14a** is flexible in solution. According to NOESY NMR spectroscopy, the preferred relative arrangement of the proline rings and the aromatic moieties in solution resembles that in the crystal structure, but the secondary amide groups rotate. Independent of the actual orientation, the three aromatic rings in **14a** surround a shallow dish-shaped cavity that should be available for cation binding. Indeed, the signals of quaternary ammonium ions such as BTMA experience upfield shifts in the ^1^H NMR spectrum upon addition of **14a**, which are stronger than those caused by **13b**. In addition, **14a** proved to be more sensitive to the presence of anions. Only picrate and the hydrophobic tetrakis[3,5-bis(trifluoromethyl)phenyl]borate (BARF) anion have no propensity to interact with **14a**, while iodide, which does not bind to **13b**, and particularly tosylate both interact with the proline-containing cyclopeptide. The reason for the ability of **14a** to bind even the weakly coordinating iodide anion is likely that the conformational reorganization required for anion binding is energetically less costly than in the case of **13b**. Both iodide and tosylate thus reinforce the stability of the cation complexes of **14a** for similar reasons as in the ion pair complexes of **13b**. The way both the cation and the anion are bound in the respective complexes, with the cation residing in the shallow cavity produced by the aromatic moieties and the anion hydrogen bonding to the NH groups, is nicely reflected in the crystal structure of the *N*-methylquinuclidinium iodide complex of **14a** (Figure 8c).

An intramolecular conformational control over the preferred conformation of **14a** was achieved by introducing substituents into the 4-positions of the aromatic moieties [75,76]. Some of the substituents tested (e.g., methyl) reduce the cation affinity likely because they prevent the respective cyclopeptide to adopt a conformation suitable for cation complexation. Other substituents, particularly those able to form intramolecular hydrogen bonds to the neighboring NH group, such as the methoxy groups in **14b** and the methoxycarbonyl groups in **14c**, cause a pronounced increase of cation affinity. These substituents rigidify the cyclopeptide by preventing the secondary amide groups from rotating. As a consequence, receptor preorganization is improved, and cation binding becomes stronger. Complex stability likely additionally benefits from the increase of the negative electrostatic potential surface along the cavity caused by the substituents [76]. The *K*_a_ of the BTMA picrate complex thus increases in CDCl_3_ from 1200 M^−1^ for **14a** to 2700 M^−1^ for **14b**, and further to 10,800 M^−1^ for **14b**.

**Scheme 5:**
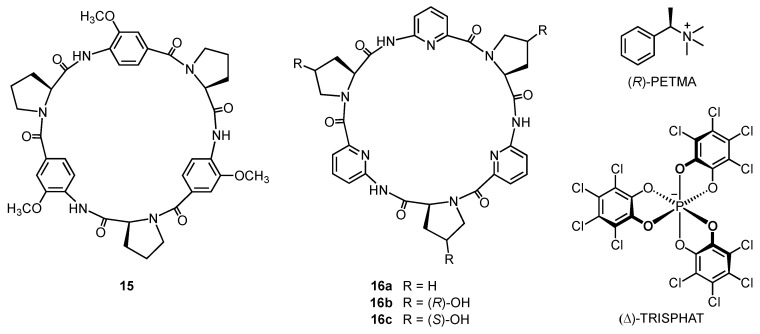
Structures of cyclopeptides **15** and **16a**–**c**.

That cation binding is not only due to cation-π interaction between the aromatic subunits of the cyclopeptides and the quaternary ammonium ions but also mediated to some extent by the carbonyl groups, which surround the cavity and contribute to the negative electrostatic potential [77], became apparent when characterizing the structure and binding properties of **15** (Scheme 5) [78]. This cyclopeptide contains 4-aminobenzoic acid residues, rendering it formally a paracyclophane, but has otherwise a similar structure as **14b**. NMR spectroscopy indicated that **15** also adopts on average a *C*_3_ symmetric conformation in DMSO or CDCl_3_, which is closely related to the preferred conformation of **14b**. The substitution pattern of the aromatic residues causes the carbonyl groups in **15** to diverge from the cavity center, however, rather than being arranged in a converging matter as in **14b**. As a consequence, **15** turned out to be unable to bind cations, not even cations that are strongly bound to **14b**. It should be noted that a cyclic hexapeptide containing alternating glycine and 2-aminobenzoic acid subunits also adopts bowl-shaped conformations according to work of the Feigel group, but the binding properties of this cyclopeptide were not evaluated [79].

The substituted cyclopeptides **14b** and **14c** are also able to differentiate the enantiomers of chiral quaternary ammonium ions [80]. The complex of **14b** with the *R*-enantiomer of *N*,*N*,*N*-trimethyl-1-phenylethylammonium (PETMA) picrate is more stable by a factor of ca. 1.5 than that of the respective *S*-enantiomer, for example (*K*_a_(*R*) = 1550 M^−1^, *K*_a_(*S*) = 1030 M^−1^ in 0.1 vol% DMSO-*d*_6_/CDCl_3_). When using diastereomeric ion pairs as guests containing PETMA and the chiral TRISPHAT anion (Scheme 5), the salt containing *R*-PETMA and *Δ*-TRISPHAT form the most stable complex, while the least stable complex is formed with *S*-PETMA/*Λ*-TRISPHAT *K*_a_(*R*/*Δ*) = 5210 M^−1^, *K*_a_(*S*/*Λ*) = 2080 M^−1^ in 0.1 vol% DMSO-*d*_6_/CDCl_3_) [81]. Moreover, the stability constants of the complexes of **14b** with *R*-PETMA/*Δ*-TRISPHAT and *S*-PETMA/*Δ*-TRISPHAT differ more strongly than those of the corresponding *Λ*-TRISPHAT salts. This influence of the anion was attributed to the fact that complex formation involves binding of the whole ion pair, allowing structural effects of the anion to affect complex stability.

The substituted cyclopeptides **14b** and **14c** are unable to engage in specific interactions with anions because the substituents prevent the NH groups from forming intermolecular hydrogen bonds [76]. Thus, the stability of the cation complexes does not increase when exchanging the counterion from picrate, which does not interact with **14a**, for anions that bind to the unsubstituted peptide such as iodide or, even more strongly, tosylate, but rather decreases in the same direction. This dependence mirrors the counterion effect on the stability of complexes of quaternary ammonium ions with cyclophanes or calix[4]arenes, and has been attributed to polarization effects in the ion pair [82] and/or the strength of ion pairing [83].

The anion affinity of such cyclopeptides cannot only be turned off by introducing substituents into the aromatic subunits that stabilize a diverging arrangement of the NH groups, it can also be improved by exchanging the 3-aminobenzoic acid subunits for 6-aminopicolinic acid residues, affording cyclopeptide **16a** (Scheme 5) [84]. Repulsive interactions destabilize a conformation of **16a** in which the ring nitrogen and carbonyl oxygen atoms of the neighboring amide group are arranged in close proximity. As a consequence, **16a** preferentially adopts a conformation in polar aprotic or protic solvents with a converging arrangement of the NH protons, which is well preorganized for anion binding. Accordingly, the stability of the tosylate complex in DMSO-*d*_6_ increases from 150 M^−1^ for the flexible and not preorganized **14a** to 4500 M^−1^ for **16a** under the same conditions.

**Figure 9:**
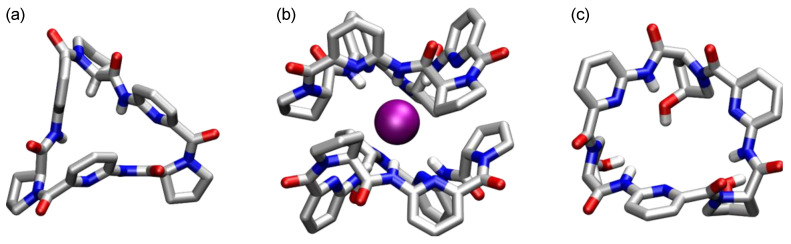
Crystal structures of **16a** (CSD Entry: TIFKIC) (**a**), the sandwich-type iodide complex of **16a** (CSD Entry: TIFKOI) (**b**), and of **16c** (CSD Entry: XITDIN) (**c**). Solvent molecules, counterions, and protons, except acidic ones, and the H(α) protons in (**a**) are omitted for reasons of clarity.

The crystal structure of **16a** (Figure 9a) shows that the conformation of this cyclopeptide differs strongly from those of **13a** or **14a** since the tertiary amide groups adopt *cis*-conformations, causing an orientation of the aromatic subunits almost parallel to the *C*_3_ axis of the macrocycle. Thus, the cavity in which cation binding takes place in the case of **13a** is lost, while the anion binding site is surrounded by the apolar proline rings. The NH groups are located at the bottom of the cavity that also features a converging arrangement of the proline H(α) protons. Whether these protons contribute to anion binding as hydrogen bond donors is unclear, but their proximity to the binding site conveniently allows complex formation to be studied by ^1^H NMR spectroscopy, because the deshielding of these protons by an anion bound in close proximity can even be followed in protic solvents in which the NH signals are not visible as a result of H/D exchange. In these solvents, complex formation also takes place. For example, benzenesulfonate forms a 1:1 complex with **16a** in 80 vol% water/methanol with a *K*_a_ of 44 M^−1^ [84]. This stability constant may seem small, but it is remarkable that a neutral receptor whose interactions with the substrate are based on hydrogen bonding forms a complex in this highly competitive medium at all. The most likely reason is that the desolvation of the receptor cavity contributes with a thermodynamically favorable term to complex formation. Indeed, the crystal structure of **16a** contains three water molecules bound to the NH groups of the cyclopeptide whose arrangement suggests that hydrating water molecules are not able to form the maximum number of hydrogen bonds in solution. The release of these water molecules thus allows them to gain hydrogen bonds, which is thermodynamically favorable [85].

Anions lacking a large organic residue such as halides and sulfates also bind to **16a**, but with a different binding mode. These anions prefer to form sandwich-type 2:1 complexes, in which an anion resides between two cyclopeptide rings [84]. The crystal structure of the iodide complex of **16a** (Figure 9b) illustrates this arrangement. In this complex, the anion forms six hydrogen bonds to NH groups. Both cyclopeptide rings are almost perfectly shape complementary, allowing them to interdigitate, which brings the apolar faces of the proline rings into almost van der Waals contact. The formation of such complexes in aqueous solution is thus accompanied by an extensive desolvation of hydrophobic surfaces, which causes the hydrophobic effect to contribute to complex formation. An indication for the importance of this effect is that the equilibrium constants associated with the binding of the second cyclopeptide ring to the initially formed 1:1 complexes are typically more than two orders of magnitude larger than those of the first binding step [86]. Complex formation thus proceeds in a highly cooperative manner, demonstrating that the release of solvent molecules associated with the formation of the sandwich complex and the dispersion interactions between the proline rings that thus become possible contribute strongly to the overall stability [87]. Together with the shielding of the anion from the surrounding medium in the final complex, which strengthens hydrogen bonding, these effects cause the halide and sulfate complexes of **16a** to reach log *K*_a_ values in 80 vol% water/methanol of up to 5.2 [84]. Halide affinity increases in the order chloride < bromide < iodide, likely because the dehydration of these anions becomes easier in the same direction and the chloride anion is too small to interact with all six NH groups of the two cyclopeptide rings simultaneously.

Based on the structural motif of the sandwich complexes of **16a**, linkers were introduced between the two cyclopeptide moieties to obtain 1:1 complexes [88]. In this context, the effects of linker structure on anion affinity of the corresponding bis(cyclopeptides) were investigated. Suitable linkers were identified using molecular modeling [89] and dynamic combinatorial chemistry [90]. In addition, bis(cyclopeptides) with more than one linker were prepared [91,92], of which some proved to possess almost nanomolar sulfate affinity in water/acetonitrile mixtures [93]. The typically reduced water solubility of such bis(cyclopeptides) was addressed by introducing solubilizing groups into the aromatic subunits and the linker [94], and the corresponding bis(cyclopeptide) provided deep insight into general principles of anion binding in water [95]. Finally, bis(cyclopeptides) that allow the optical sensing of anions in solution were also prepared [96]. This work is outside the scope of this review because it builds on the anion affinity of **16a** and does not involve changing the preferred cyclopeptide conformation. Interested readers are therefore referred to another review [97].

Two additional monocyclic peptides are worth mentioning, **16b** and **16c** (Scheme 5) that contain (2*S*,4*R*)- and (2*S*,4*S*)-4-hydroxyproline subunits, respectively [86]. The cyclopeptide **16b** is water soluble and adopts a conformation resembling that of **16a**. In contrast to **16a**, it only forms 1:1 complexes with inorganic anions, likely because the hydroxy groups prevent the formation of sandwich complexes for steric reasons and reduce the extent to which the hydrophobic effect contributes to complex formation because of the hydration of the hydroxyproline units. The stability constants of the 1:1 halide and sulfate complexes of **16b** in 80 vol% water/methanol are, however, comparable to the stability constants associated with the formation of the 1:1 complexes of **16a** under the same conditions [86].

The hydroxy groups in **16c** cause this peptide to exist in two *C*_3_ symmetric conformations in solution, an all-*cis* and all-*trans* conformation, that interconvert slowly on the NMR time scale [86]. In the solid state, only the all-*trans* conformation was observed. The respective crystal structure (Figure 9c) shows that the stabilization of the *trans*-amides is caused by intramolecular hydrogen bonds between the OH and NH groups.

**Scheme 6:**
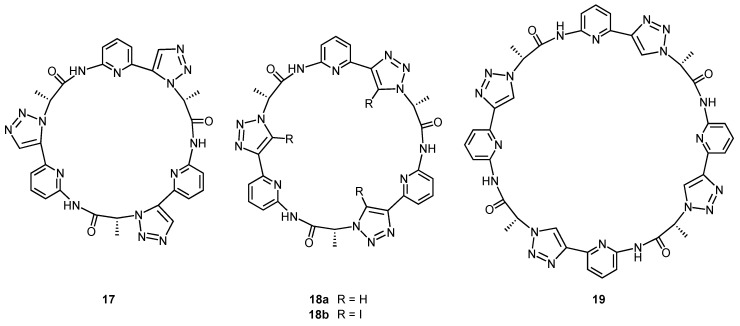
Structures of the cyclic pseudopeptides **17**–**19**.

**Figure 10:**
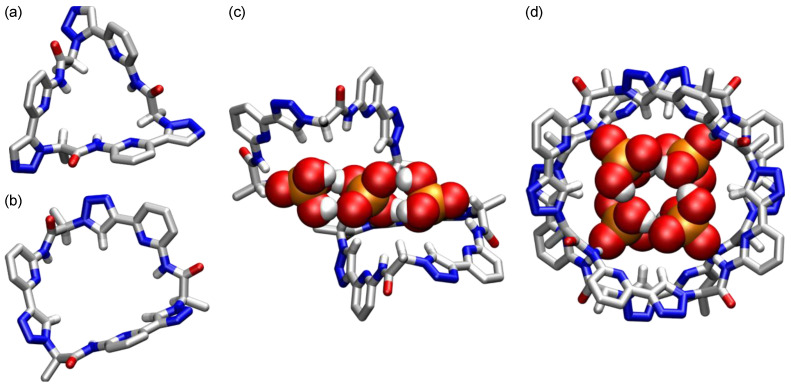
Crystal structures of **17** (CSD Entry: HADVIT) (**a**), **18a** (CSD Entry: EYUKUG) (**b**), the 2:3 dihydrogenphosphate complex of **18a** (CSD Entry: EYUTEZ) (**c**), and the 2:4 dihydrogenphosphate complex of **19** (CSD Entry: XAZQAT) (**d**). Solvent molecules, counterions, and protons, except acidic ones, and the H(α) and the triazole H(5) protons in (**b**–**d**) are omitted for reasons of clarity.

Further structural modifications of these cyclopeptides mainly involved the introduction of substituents into the aromatic subunits to induce affinity for carbohydrates [98,99] or amino acids [100], or into the proline rings to mediate sulfate binding [101]. In addition, the replacement of amide groups along the ring by other linking units was also explored. The motivation in this context was to investigate whether it is possible to transfer the anion affinity of **16a** to other macrocyclic receptors by mimicking the structural parameters that control the preferred cyclopeptide conformations. Two parameters are important in this respect: the effect of the heterocyclic units on the orientation of the NH groups and the existence of *cis*-amides at the proline residues. Since a widely used strategy in the design of peptidomimetics involves the exchange of *cis*-amides in a peptide backbone with 1,5-disubstituted 1,2,3-triazole residues, which closely mimic *cis*-amides in terms of structure and electronic properties [102], the cyclic pseudopeptide **17** (Scheme 6) was identified as a possible analog of **16a** [103]. This pseudopeptide indeed prefers a conformation in solution and the solid state with a converging arrangement of the protons at the NH groups and the stereogenic centers according to NMR spectroscopy and X-ray crystallography (Figure 10a). The pseudopeptide therefore also binds halides and sulfate in methanol and water/methanol mixtures. While the intrinsic anion affinity seems to be even higher than that of **16a**, **17** has a less pronounced tendency to form 2:1 complexes, likely because of structural reasons and a reduced thermodynamic gain associated with the desolvation of the small methyl groups in the side chains.

The 1,4-disubstituted 1,2,3-triazole units in the cyclic pseudopeptide **18a** (Scheme 6) acts as surrogates for *trans*-amides [104]. In this pseudopeptide, the NH and the triazole protons converge because an arrangement with the nitrogen atoms of the five and the six-membered subunits pointing in the same direction is disfavored. Accordingly, **18a** is also well preorganized for anion binding, even featuring twice the number of hydrogen bond donors than **17**. Conformationally, however, **18a** resembles the all-*trans* cyclopeptides with 3-aminobenzoic acid subunits such as **14a**, as illustrated by the crystal structure (Figure 10b). As a consequence, **18a** is insoluble in aqueous solvent mixtures so that binding studies had to be performed in acetone/DMSO or DMSO [104]. In acetone/DMSO, **18a** binds to halides and various oxoanions, but in the more competitive DMSO, only the binding of sulfate and dihydrogenphosphate anions is retained. Since the triazole units cause the cavity diameter of **18a** to be larger than that of **14a**, higher complexes are mostly formed, involving more than one anion and/or more than one pseudopeptide ring. For example, the crystal structure of the dihydrogenphosphate complex of **18a** demonstrates the sandwiching of a chain of three hydrogen-bonded dihydrogenphosphate anions between two pseudopeptide rings (Figure 10c). This relatively unspecific binding can be overcome by replacing the 1,2,3-triazole units in **18a** with 5-iodo-1,2,3-triazoles [105]. The iodine atoms in the corresponding pseudopeptide **18b** decrease the cavity diameter and mediate anion binding by halogen bonding. Accordingly, **18b** is able to bind halides in the form of 1:1 complexes, with the chloride complex, for example, having a log *K*_a_ of 3.4 in 2.5 vol% water/DMSO. Under similar conditions, the parent pseudopeptide **18a** does not interact with halides, showing the advantage of the iodide atoms.

Counterintuitively, the larger cyclooctapeptide analog **19** forms structurally better-defined complexes than **18a** [106]. Sulfate binding involves the formation of a 1:1 complex with a moderate log *K*_a_ of 3.1 in 2.5 vol% water/DMSO, in which one pseudopeptide ring likely folds around the anion. The complexation of dihydrogenphosphate or dihydrogenpyrophosphate is more interesting since it involves the sandwiching of a hydrogen-bonded dimer of dihydrogenpyrophosphate or a hydrogen-bonded cyclic tetramer of dihydrogenphosphate anions between two pseudopeptide rings. The crystal structure of the latter complex is depicted in Figure 10d to illustrate this arrangement. No major conformational reorganization of **19** is required for the formation of these complexes. In addition, the NH and CH hydrogen bond donors of **19** make extensive contacts to the oxygen atoms of the bound anions, causing these complexes to be sufficiently stable to even survive the transfer into the gas phase where they can be detected by mass spectrometry. The receptor-induced stabilization of hydrogen-bonded anion aggregates such as those observed in the complexes of **19** is not unprecedented [107]. The efficient binding of **19** to such aggregates allows the pseudopeptide to differentiate two structurally similar anions, however, of which only the protonated phosphate-derived species can form aggregates and the fully deprotonated sulfate cannot. Accordingly, recognizing anion aggregates instead of simple anions could represent a promising concept to improve the selectivity of anion receptors, particularly for biologically relevant phosphates.

**Figure 11:**
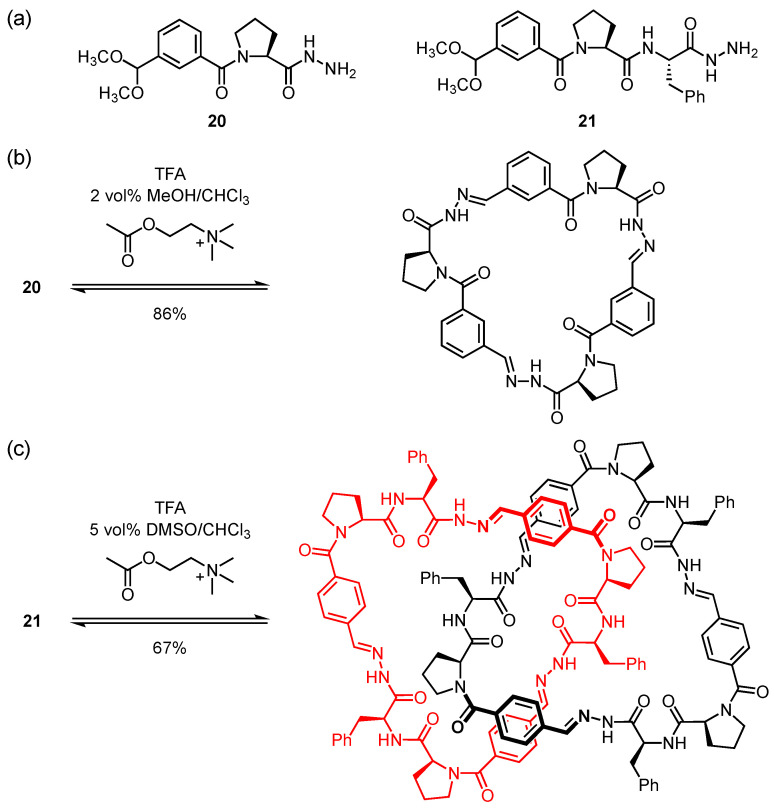
Structures of building blocks **20** and **21** (**a**), acetylcholine-templated synthesis of a macrocyclic trimer from **20** (**b**), and of a [2]catenane comprising two macrocyclic trimers from **21** (**c**).

The Sanders group used amino acid-containing building blocks to access macrocyclic pseudopeptides by using the concepts of dynamic combinatorial chemistry [58]. The respective building blocks, examples are **20** and **21** (Figure 11a), contain a hydrazone group at one end of the chain and an acetal group at the other. Treating these compounds with an acid induces the cleavage of the acetal group and the concomitant release of the aldehyde, which subsequently reacts with the hydrazides under the acidic conditions to afford acyl hydrazones. Once the respective reaction mixtures reach thermodynamic equilibrium, they usually contain a mixture of macrocycles in ratios reflecting the thermodynamic stability of the individual products under the chosen conditions. The exchange of the building blocks can be stopped by neutralization and the products can then be isolated. As long as the exchange proceeds, however, product distribution can be influenced by, for example, adding a suitable binding partner to the solution. These compounds potentially template the formation of species in the library with which they form a complex, even of products that do not exist in the absence of the template. The stabilization induced by complex formation leads to the amplification of the corresponding products, allowing not only the identification but also the subsequent preparation of the receptor.

In an early application of this concept, the Sanders group showed that treating **20** in chloroform with trifluoracetic acids initially induces the formation of a complex mixture of macrocyclic pseudopeptides, comprising mainly the cyclic dimer, trimer, and tetramer and smaller amounts of higher oligomers [108]. Once thermodynamic equilibrium is reached, the dimer dominates in the mixture, but the trimer is also present. The addition of acetylcholine causes re-equilibration, leading to an increase of the relative amount of the cyclic trimer from 11% to 86% (Figure 11b). Binding studies with the isolated product were complicated by a slow conformational equilibrium of the macrocycle. These studies nevertheless provided clear evidence for an interaction, likely by CH···O hydrogen bonds between the N–CH protons of acetylcholine and the carbonyl oxygen atoms of the pseudopeptide. A *K*_a_ of 230 M^−1^ was determined for the respective complex in 5 vol% CD_3_OD/CDCl_3_.

When treating **21** with acid in 2 vol% MeOH/CHCl_3_, a series of macrocycles differing in ring size was observed [109]. The addition of a lithium salt or, to a lesser extent, a sodium salt, induces the amplification of the cyclic trimer. Independent binding studies demonstrated that this trimer binds to lithium ions with a log *K*_a_ of 4.6 in this solvent mixture, likely by interacting with the carbonyl groups of the cyclic pseudopeptide. Complex formation leads to a conformational stabilization of the otherwise flexible receptor. Treating the dynamic library obtained from **21** with quinine induces the amplification of the macrocyclic tetramer, while quinidine templates the formation of the respective dimer [110]. Remarkably, using acetylcholine as template affords a [2]catenane comprising two interlocked macrocyclic trimers as the major product (Figure 11c) [111]. With a log *K*_a_ of 7.1, this catenane has a remarkable affinity for acetylcholine in 5 vol% DMSO/CHCl_3_. The exact binding mode is not entirely clear, but acetylcholine likely inserts into a binding pocket located between the two rings.

Luis, Alfonso, and coworkers investigated the structures, self-assembly, and recognition properties of cyclic pseudopeptides derived from suitable diamines [7]. These macrocycles were obtained by coupling *N*-protected α-amino acids, mostly l-valine or l-phenylalanine, to suitable α,ω-diamine precursors. After deprotection, the resulting bis(amino acid) derivatives were reacted with 1,3- or 1,4-bis(bromomethyl)benzene or analogous building blocks to obtain the corresponding [1 + 1] macrocyclization products. When reacting the diamine precursors with aromatic dialdehydes under reductive amination conditions, larger [2 + 2] macrocycles are formed.

**Scheme 7:**
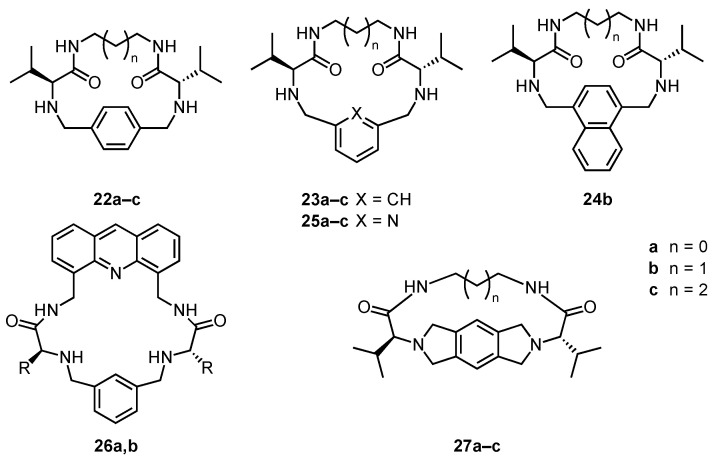
Structures of the cyclic pseudopeptides **22**–**27**.

The efficiency of the macrocyclization that affords the [1 + 1] pseudopeptides **22a**–**c** and **23a**–**c** (Scheme 7) in typical yields of circa 60% without the need of high dilution conditions was attributed to the conformational stabilization of the intermediate resulting from the first nucleophilic substitution by an intramolecular hydrogen bond, which facilitates the ring closure in the second step [112,113].

**Figure 12:**
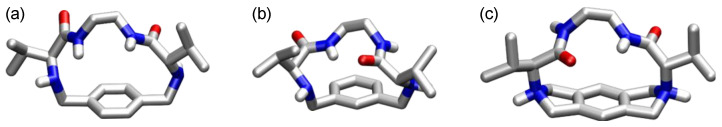
Crystal structures of **22a** (CSD Entry: UHUSUL) (**a**), **23a** (CSD Entry: UHUSOF) (**b**), and **27a** (CSD Entry: XUYBAX) (**c**). Solvent molecules, counterions, and protons, except acidic ones, are omitted for reasons of clarity.

Crystal structures of **22a** and **23a** (Figure 12a,b) demonstrated that the aliphatic chains fold around the aromatic residues in a conformation that is stabilized in the case of **23a** by an intramolecular hydrogen bond [113]. Detailed conformational studies of these [114] and the related naphthyl-containing cyclic pseudopeptides **24a**,**b** (Scheme 7) [115] showed that the macrocycles are flexible in solution and that the aromatic rings rotate (or flip in the case of the naphthyl residues). The rate of conformational interconversion depends on ring size, the nature of the amino acid side chain, and the strength of the intramolecular hydrogen bonds, the latter of which is a function of solvent polarity.

Binding studies mainly addressed the interactions of these pseudopeptides with amino acids, although it was also shown that the pyridyl-containing pseudopeptides **25a**–**c** (Scheme 7) coordinate to Ag^+^ ions through the ring nitrogen atom and the carbonyl groups [116]. The naphthyl-containing pseudopeptide **24b** was shown to accept protons from *N*-protected amino acids [117,118]. The ions formed in this way subsequently pair in dichloromethane, and since **24b** contains two basic amino groups, up to two amino acids can be bound in this way. The stepwise binding is noncooperative since the second amino acid is bound with a considerably lower binding constant than the first. Strongest binding was observed for Z-Phe-OH, which was attributed to aromatic interactions between the naphthyl group of the pseudopeptide and the benzene ring in the side chain of the substrate. Phenylalanine recognition was also moderately enantioselective (*K*_a_(Z-l-Phe-OH) = 193 M^−1^, *K*_a_(Z-d-Phe-OH) = 214 M^−1^, with both stability constants referring to the formation of the 1:1 complex in dichloromethane at 278 K).

The acridine-containing cyclic pseudopeptide **26a**,**b** (Scheme 7) binds unprotected tryptophane in aqueous 1 M sulfuric acid [119]. Under these conditions, both the host and the guest are fully protonated and therefore cationic. The addition of tryptophane to the solution of **26a**,**b** nevertheless causes the quenching of the receptor fluorescence, which suggests an interaction. Complex formation was attributed to aromatic interactions between the tryptophane indole and the receptor acridine moiety. In addition, computational results provided evidence that the two cationic species are held together by hydrogensulfate anions that are also present in the medium. The acridine-containing pseudopeptides are only fluorescent after full protonation, which initially involves the two basic amino groups in the ring and finally the nitrogen atom in the acridine moiety. Accordingly, the fluorescence can be turned on by treating **26a** with acids in chloroform [120]. These investigations showed that the fluorescent response of **26a** strongly depends on the nature of the acid, with phosphoric acid producing a much larger increase of the emission intensity than sulfuric acid. Other acids are almost ineffective. The pronounced increase of receptor fluorescence in the presence of phosphoric acid was attributed to the interactions of the triply protonated form of the receptor with a dihydrogenphosphate anion.

The conformationally constrained pseudopeptides **27a**–**c** (Scheme 7) were obtained by reacting a bis(valine)-derived diamine with 1,2,4,5-tetrakis(bromomethyl)benzene [121]. The macrocyclization in this case benefits from kinetic template effects of anions, with bromide being more efficient than iodide, while chloride proved to be unsuitable. A crystal structure showed that the aliphatic chain folds around the planar hexahydropyrrolo[3,4-*f*]isoindolo moiety in the dihydrochloride of **27a** in a similar manner as in the more flexible analogs (Figure 12c). This pseudopeptide was shown to mediate the transformation of epoxides into the corresponding cyclic carbonates when reacted in the absence of a solvent with a tetrabutylammonium halide and carbon dioxide at 100 °C [122].

**Figure 13:**
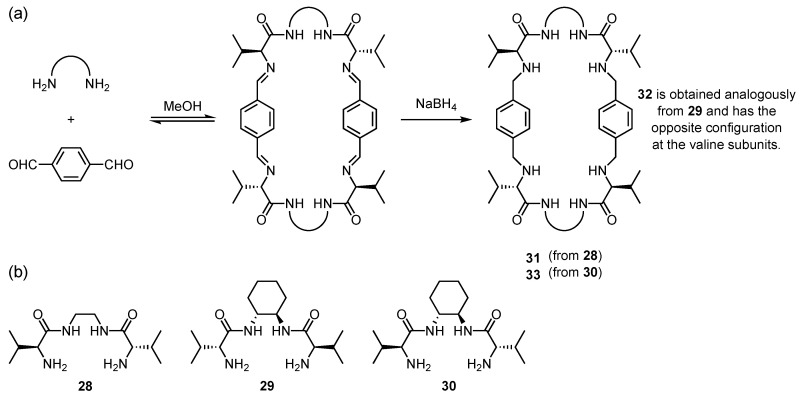
Strategy of the [2 + 2] macrocyclization by reacting diamines with flanking amino acid subunits with terephthalaldehyde via an intermediate tetraimine that is reduced to afford the stable tetraamine product (**a**), and structure of building blocks **28**, **29**, and **30** that afford the respective macrocyclic pseudopeptides **31**, **32**, and **33** (**b**).

The efficiency of the synthesis of the larger [2 + 2] macrocycles proved to depend sensitively on the structural parameters of the building blocks. For example, treating the diamine **28** or **29** (Figure 13b) derived from 1,2-diaminoethane or (1*R*,2*R*)-1,2-diaminocyclohexane, respectively, with terephthalaldehyde only affords complex product mixtures without notable amounts of macrocyclic products [123,124]. Building block **30**, on the other hand, which differs from **29** in the configuration of the amino acid subunits (*S* in **30** instead of *R* in **29**) leads to the respective tetraamine **33** after reduction of the intermediate tetraimine in a yield of 67% (Figure 13a).

**Figure 14:**
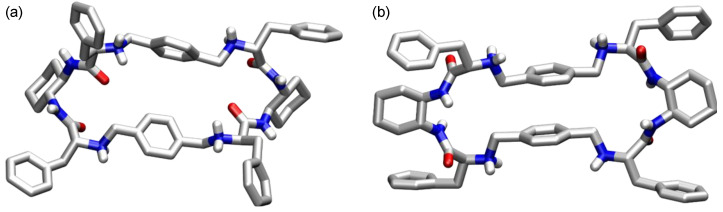
Crystal structures of the phenylalanine-derived analogs of **32**·4HCl (CSD Entry: QOJREN) (**a**) and **33**·4HCl (CSD Entry: EHEMAG) (**b**). Solvent molecules, counterions, and protons, except acidic ones, are omitted for reasons of clarity.

The rigidity of this diamine decreases the entropic cost resulting from the loss of conformational freedom during macrocyclization, thus favoring the reaction. Building block **28** is too flexible, and **29** with the mismatched relative configuration of the subunits is unsuitable because it does not arrange the reacting amino groups in a manner required for the formation of a strained-free product. If, however, the reaction between **29** and terephthalaldehyde is performed in the presence of terephthalate, the macrocyclization product **32** is obtained in 50% yield [125]. Under these conditions, macrocyclization is mediated by a template effect of the added dicarboxylate, which causes the proper preorganization of the product precursor. Template effects of aromatic dicarboxylates can also be used to induce the formation of **31**, derived from the flexible diamine precursor **28** [126,127]. To illustrate the conformation of the products, the crystal structures of analogs of **32** and **33** containing benzyl instead of isopropyl groups in the amino acid subunits are shown in Figure 14.

The pseudopeptide **33** was shown to bind *N*-protected dipeptides in chloroform [128]. Like in the case of the smaller [1 + 1] macrocycles, complex formation involves the initial proton transfer from the carboxylic acid group of the dipeptide to an amino group of the receptor. The ions formed in this way then pair, and since the dipeptide effectively fills the available cavity, further protonation of the remaining amino groups and the formation of higher complexes does not take place. Among the dipeptides tested, **33** binds most efficiently to Z-Phe-Phe-OH with a *K*_a_ of 215 M^−1^ in 1 vol% MeOH/CDCl_3_. These [2 + 2] macrocycles also self-assemble in solution and the solid state [129,130].

Macrocycles containing a combination of amino acid and aromatic subunits have also been used as optically active ligands for the nickel-catalyzed oxidation of alkenes [131], as ligands for transition metal ions [132], or as anion receptors [133,134].

## 6. Cyclic Peptides Containing Five-Membered Heterocyclic Subunits

Five-membered heterocyclic subunits can be found in a number of natural cyclopeptides. Thiazole or oxazole residues are particularly common as they (bio)synthetically originate from cysteine, serine, and threonine. These and other heterocyclic rings have also been incorporated into synthetic macrocyclic peptides to improve metabolic stability, activity, and selectivity for potential pharmaceutical applications. A comprehensive overview is beyond the scope of this article and interested readers are therefore referred to a recent review [135]. In the following, a selection of cyclopeptides is presented that can serve as ligands in metal complexes or as receptors.

**Figure 15:**
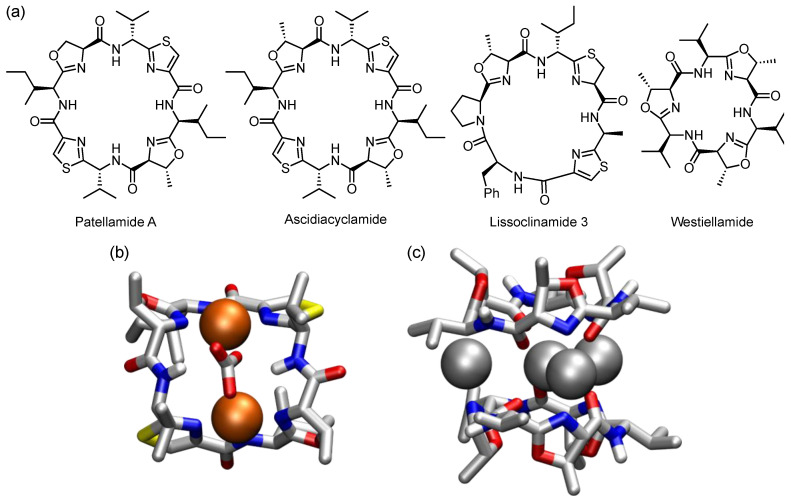
Examples of natural cyclopeptides containing oxazoline, thiazoline, or thiazole residues (**a**), and crystal structures of the dicopper(II) complex of ascidiacyclamide (CSD Entry: POHKOM) (**b**), and the tetrasilver(I) complex of westiellamide (CSD Entry: WICZAJ) (**c**). Solvent molecules, counterions, and protons, except acidic ones, are omitted for reasons of clarity.

The sea squirt *Lissoclinum patella* accumulates transition metals ions such as Cu^2+^ up to ten thousand times the concentration found in the local environment [136]. At the same time, this and other marine organisms are rich sources of cyclopeptides containing oxazoline, thiazoline, or thiazole subunits such as patellamides, lissoclinamides, ascidiacyclamides, and westiellamides, some of which have structures reminiscent of those of tetrapyrrole-based ligands (Figure 15a) [137].

It was suggested that these cyclopeptides could be the reason for the ability of the marine organisms in which they occur to sequester and transport metal ions [138], and evidence for the ability of these cyclopeptides to act as ligands for transition metals has indeed accumulated over the years. Ascidiacyclamide, for example, was shown to form a dicopper(II) complex in which the cyclopeptide adopts a saddle-shaped conformation to accommodate the two metal ions (Figure 15b) [139]. Each ion interacts with two nitrogen atoms of adjacent heterocyclic subunits. Amidate groups act as further coordination sites, and both copper ions are connected via a carbonate ion, rendering the complex overall neutral. Another example for such a metal complex is the tetrasilver(I) complex of westiellamide, in which four silver ions are sandwiched between two cyclopeptide rings (Figure 15c) [140]. The central ion interacts with six carbonyl oxygen atoms, while the other three ions reside between the nitrogen atoms of pairs of oxazoline residues.

**Scheme 8:**
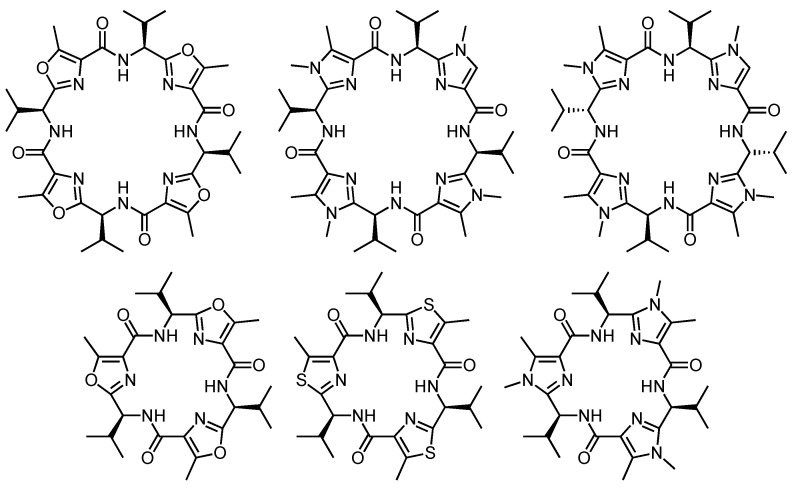
Examples of patellamide and westiellamide analogs investigated in the Comba group as ligands for transition metals, mainly copper(II).

Extensive work, also involving the use of structural analogs of the natural products, was performed in the Comba group to gain detailed insight into the coordination chemistry of such cyclopeptides and their biological functions [141]. A selection of compounds investigated in this context is shown in Scheme 8. The investigations involved typical methods of coordination chemistry, such as the characterization of the redox chemistry of the complexes by cyclic voltammetry in addition to structural investigations and the characterization of complex stability [142,143,144,145]. Since this work falls more into the domain of inorganic coordination chemistry than supramolecular chemistry, it will not be described in detail here.

**Figure 16:**
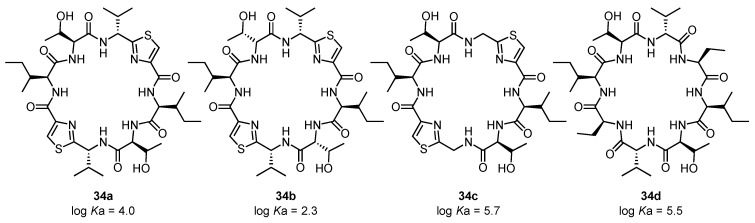
Structures of the ascidiacyclamide analogs **34a**–**d**. The Ca^2+^ affinity in 5 vol% water/acetonitrile is specified below each compound with the respective log *K*_a_ value.

Patellamides and related cyclopeptides also have a weak affinity for Ca^2+^ ions that interact with the oxygen atoms along the ring. The characterization of the analogs **34a**–**d**, in which two or even all four heterocyclic subunits are replaced by acyclic α-amino acids, showed that their Ca^2+^ affinity in 5 vol% water/acetonitrile is mostly higher than that of ascidiacyclamide (log *K*_a_ = 2.8) (Figure 16) [146]. This increase is not simply an effect of the larger number of carbonyl groups in macrocycles with more α-amino acids, as demonstrated by the similar Ca^2+^ affinities of ascidiacyclamide (four carbonyl groups) and **34b** (six carbonyl groups), or **34c** (six carbonyl groups) and **34d** (eight carbonyl groups). The high affinities of **34c** and **34d** were rather attributed to the flexibility of these cyclopeptides, which enables them to adopt conformations well suited for metal ion coordination [147].

**Figure 17:**
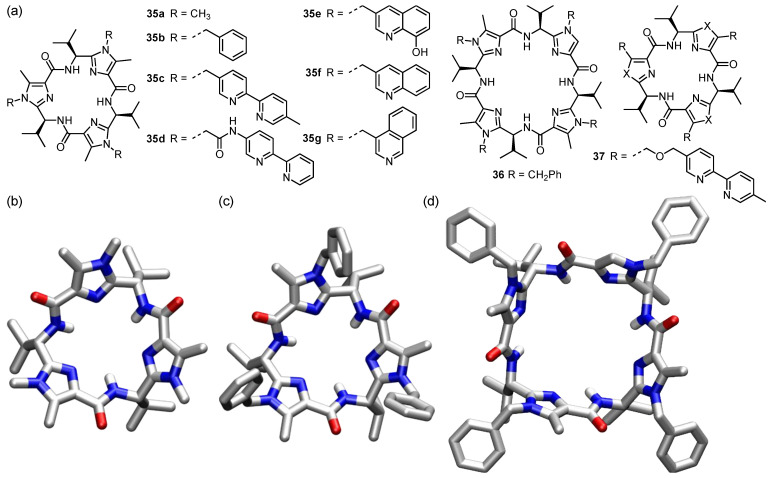
Structures of cyclopeptides **35a**–**g**, **36**, and **37** (**a**), and crystal structures of **35a** (CSD Entry: LUBKEY) (**b**), **35b** (CSD Entry: HAHNEK) (**c**), and **36** (CSD Entry: XIBDES) (**d**). Solvent molecules, and protons, except acidic ones, are omitted for reasons of clarity.

Work in the Haberhauer group concentrated on analogs of the *C*_3_ symmetric westiellamide, but also involved the use of smaller and larger macrocycles containing two or four heterocyclic subunits (Figure 17a) [148]. Mostly imidazole-containing cyclopeptides were used in this context that allow further functionalization by attaching substituents to ring nitrogen atoms [149,150,151,152,153,154]. Crystal structures of the *C*_3_ symmetric parent cyclopeptide **35a** (Figure 17b) indicated that this compound is relatively rigid, adopting a cone-shaped structure in which all imidazole units are tilted into one direction [150]. The lone pairs of the heterocyclic nitrogen atoms and the NH protons point toward the center of the macrocycle, and the three isopropyl residues reside along the narrower cavity rim in pseudoaxial positions.

It was expected that steric effects of the isopropyl groups would direct the substituents attached to the imidazole residues to the opposite side of the ring, which was indeed observed [155]. In the crystal structure of **35b**, for example, all three phenyl groups point into the same direction, lining a hydrophobic cavity (Figure 17c). The arrangement is similar in the larger analog **36**, although the phenyl groups diverge in this case so that the structure does not provide evidence for a binding pocket (Figure 17d) [152].

These *C*_3_ symmetric cyclopeptides were decorated with tripodal ligands for transition metals such as 2,2′-bipyridine units as in the cyclopeptides **35c**,**d** [155,156,157] and **37** [158], or 8-hydroxyquinoline units as in **35e** [159]. Accordingly, these cyclopeptides form octahedral metal complexes with suitable metal ions. While **35c**,**d** and **37** prefer to bind Cu^2+^, Zn^2+^, Co^2+^, or Ni^2+^, **35e** coordinates to Fe^3+^, Al^3+^, La^3+^, or Ga^3+^. The cyclic scaffold acts as a chiral inductor in these compounds, favoring the formation of the *Λ*-configured over the *Δ*-configured metal complexes. Cyclopeptide **35c** also interacts with phloroglucinol (1,3,5-benzenetriol) with a *K*_a_ of 680 M^−1^ in 10 vol% acetonitrile/chloroform [155]. A crystal structure confirmed that phloroglucinol is bound in the respective complex to all three side arms of **35c**, forming hydrogen bonds to the ring nitrogen atoms.

**Scheme 9:**
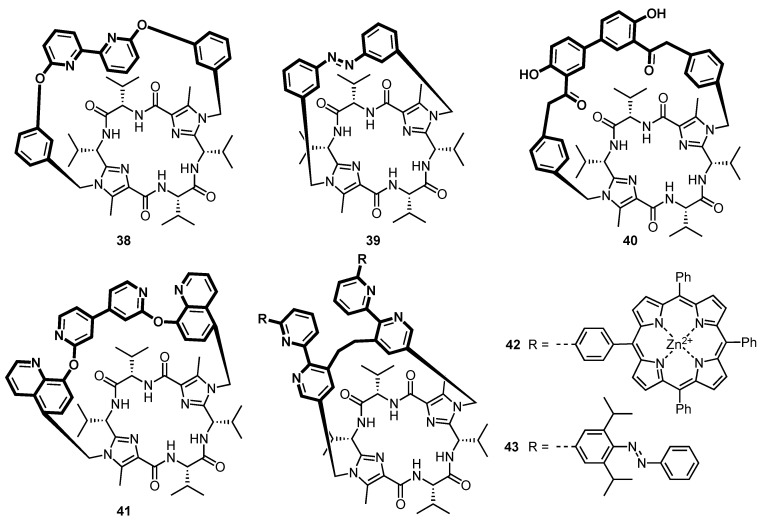
Structures of the cyclopeptide-based molecular hinges **38**, **39**, and **40**, the molecular pushing motor **41**, the molecular pendulum **42**, and the four-stroke motor **43**.

Chirality transfer from the cyclopeptide ring to appended substituents was also used to realize unidirectional motions. In the metal-free form of the so-called molecular hinge **38** (Scheme 9), for example, the 2,2′-bipyridine moiety has the anti-conformation to avoid the repulsion of the nitrogen lone pairs [160]. In this form, **38** exhibits planar chirality and prefers the *M*-configuration because of steric effects of the chiral scaffold. The addition of a Cu^2+^ salt induces a rotation around the central bipyridine bond to allow both nitrogen atoms to coordinate to the metal ion. Planar chirality is lost in this process, but when the now closed hinge is once again opened by adding a stronger copper binder, the *M*-configured cyclopeptide is restored. Since this process is stereoselective, the corresponding motion of the hinge is unidirectional. By structurally varying the cyclopeptide scaffold or using other metal ions, the amplitude of the motion can be controlled [161].

The structurally related cyclopeptides **39**–**43** (Scheme 9) feature similar unidirectional motions. In the case of **39** with the azobenzene-derived strap, light-induced *E*/*Z*-isomerization of the azobenzene moiety allows switching from the *E*-isomer, in which the azobenzene unit is planar and therefore not a stereogenic element, exclusive to the *P*-configured *Z*-azobenzene [162]. A similar effect can be induced during a redox cycle by switching the bis(phenol) unit in **40** from the reduced axial chiral form to the oxidized planar structure and back [163]. In the case of **41**, metal complexation and decomplexation allows switching between two states with opposite chirality [164]. The cyclopeptide **42** behaves like a pendulum since the two porphyrin moieties are oriented on opposite sides of the ring [165]. Metal complexation and decomplexation then causes the substituents to swing back and forth. Cyclopeptide **43** responds to different stimuli, namely, metal coordination and light [166]. It can therefore be switched between four states, each of which has a characteristic structure. The most compact one is the metal-free form with the *Z*-azobenzene unit. The sequence metal coordination and light induced *Z* → *E* isomerization leads to the most extended structure via an intermediate state. Metal decomplexation and isomerization to the *Z*-azobenzene then leads back to the compact state. This sequence was compared with the functional principle of a four-stroke motor.

With regard to binding properties, the cyclopeptides **35f** and **35g** (Figure 17) with, respectively, quinoline and isoquinoline side chains were shown to interact enantioselectively with α-chiral ammonium ions in chloroform [167]. NMR studies indicated that the ammonium ions interact with the ring nitrogen atoms of the substituents. The binding constants range between log *K*_a_ values of 2.0 and 4.5, and the stability constants of the complexes of a pair of guest enantiomers differ by a factor of circa 9 in the best case, with the respective *R*-enantiomer usually forming the more stable complex. The *K*_a_ of the complex between **35g** and (*R*)-1-phenylethylammonium perchlorate amounts to 30,000 M^−1^ in CDCl_3_, for example, while the respective *S*-enantiomer is bound with a *K*_a_ of 4500 M^−1^.

**Scheme 10:**
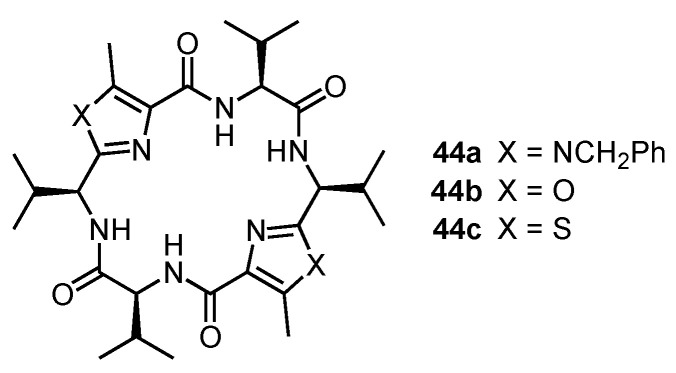
Structures of the anion-binding azole-containing cyclopeptides **44a**–**c**.

The three converging NH groups of the *C*_2_ symmetric cyclopeptides **44a**–**c** (Scheme 10) render these compounds preorganized for anion binding [168]. Anion selectivity is influenced by the basicity of the anion and its fit within the available cavity. Accordingly, halide affinity increases in the direction I^−^ < Br^−^ < Cl^−^ < F^−^. Oxoanions such as acetate and dihydrogenphosphate, which can form two hydrogen bonds to the NH groups, are most strongly bound. Although acetate is more basic than dihydrogenphosphate, the latter anion forms the more stable complexes, which was attributed to additional hydrogen bonds between the dihydrogenphosphate protons and the nitrogen atoms in the heterocyclic rings of the cyclopeptides. The less basic thiazole-containing receptor **44c** forms the most stable complexes with H_2_PO_4_^−^ (*K*_a_ = 30,000 M^−1^) and acetate (*K*_a_ = 23,400 M^−1^) in 5 vol% DMSO-*d*_6_/CDCl_3_, but anion selectivity of the more basic imidazole-containing cyclopeptide **44a** is best, since it binds H_2_PO_4_^−^ (*K*_a_ = 2640 M^−1^) ten times stronger than acetate (*K*_a_ = 270 M^−1^). Hence, the nature of the azole unity in these receptors has a distinct influence on anion selectivity.

**Scheme 11:**
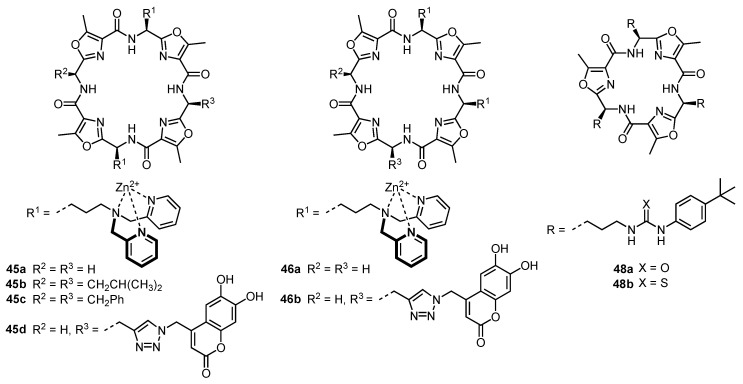
Structures of the phosphate-binding cyclopeptides **45a**–**d** and **46a**,**b** containing zinc-DPA moieties, and **48a**,**b** containing urea and thiourea groups in the side chains.

The Jolliffe group also used azole-containing cyclopeptides as anion receptors [169]. These receptors were mostly based on oxazole-containing cyclopeptides in which two zinc-dipicolylamine (DPA) moieties in distal or proximal positions serve as primary binding sites. Although additional interactions between the anions and functional groups along the cyclopeptide ring cannot be excluded, the cyclic scaffold mainly represents a versatile platform to investigate the effects of structural variations on binding properties. In this context, the length of the linkers between the DPA units and the ring and the structure of the aliphatic side chains were varied. A selection of receptors investigated in this context is shown in Scheme 11.

Indicator displacement assays showed that all of these cyclopeptides strongly bind to pyrophosphate (PPi) with apparent binding constants log *K*_a_ approaching or even exceeding 9 in aqueous HEPES buffer (5 mM, pH 7.4) [170]. Adenosine triphosphate (ATP) and adenosine diphosphate (ADP) are also bound, but often less strongly. The influence of the side chains on anion selectivity is reflected in the higher selectivity for PPi over ATP or ADP of receptor **45a** with the small methyl groups in comparison to the analogs **45b,c** with the more bulky substituents [171]. Cyclopeptide **45c** with benzyl groups forms a more stable complex with ATP (log *K*_a_ = 8.6) than with PPi (log *K*_a_ = 8.4), which was attributed to stabilizing interactions between the aromatic residues of the host and the nucleotide. Shifting the zinc–DPA units from a distal to a proximal arrangement as in **46a** also affects anion selectivity [169].

**Figure 18:**
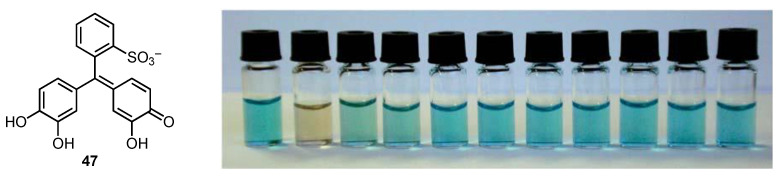
Structure of indicator **47** and picture of solutions containing equimolar mixtures of **46a** and **47** (20 μM in 5 mM HEPES buffer at pH 7.4 containing 145 mM NaCl). The vial on the left-hand side contains no additional salt while the other vials contain the sodium salts of PPi, ATP, ADP, AMP, cAMP, phosphothreonine, phosphoserine, phosphotyrosine, HPO_4_^2−^, and citrate (5 equiv each) (from left to right). The picture is reprinted from Ref. [170] with permission from the Royal Society of Chemistry.

The indicator displacement assays that were used to follow complex formation not only allowed the analytes to be detected by using simple optical methods, but often even with the naked eye. Moreover, PPi and nucleotides could be sensed selectively even in complex aqueous media such as Krebs buffer (137 mM NaCl, 5.4 mM KCl, 1.2 mM MgSO_4_, 2.8 mM CaCl_2_, 0.4 mM KH_2_PO_4_, 0.3 mM NaH_2_PO_4_, 10 mM TRIS, 10 mM glucose in water buffered to pH 7.4) [171], indicating that such assays can be used in biomedical applications. To illustrate the remarkable selectivity for PPi and the ease with which this anion can be detected, an image is shown in Figure 18 of a series of samples containing 1:1 mixtures of receptor **46a** and the indicator **47** in aqueous HEPES buffer (5 mM, pH 7.4, 145 mM NaCl) as well as different anions [170].

To avoid the use of mixtures of receptors and indicators, Zn–DPA containing cyclopeptides that contain a covalently appended fluorophore were also studied [172]. The corresponding cyclopeptide **46b** exhibits a large enhancement of the coumarin fluorescence when binding to PPi, while the fluorescence is much less strongly affected by ATP and ADP. The analogous receptor **45d** is less sensitive and selective for PPi, likely because the coumarin residue interacts with both substituents, making its displacement upon analyte binding more difficult.

Introducing suitable recognition motifs into the side chains of such cyclopeptides also afforded sulfate receptors [173]. The corresponding tris(urea) and tris(thiourea) derivatives **48a**,**b** (Scheme 11) proved to be so efficient that NMR spectroscopic binding studies in 10 vol% DMSO-*d*_6_/CDCl_3_ only allowed estimating lower limits of sulfate affinity of 10^4^ M^−1^. Other tested anions such as halides, nitrate, acetate, and dihydrogenphosphate are bound significantly less strongly. This selectivity was attributed to the ability of sulfate anions to form hydrogen bonds to both the urea and cyclopeptide NH groups simultaneously, while spherical anions and oxoanions with two or three hydrogen bond acceptors can only interact with the three urea units. The Jolliffe group also explored the effect of the length of the linker between the cyclopeptide ring and the urea groups on anion affinity [174]. In this context it turned out that sulfate affinity is retained when decreasing the linker length. Arranging the urea units closer to the cyclopeptide ring also improves the binding of anions that only weakly interact with **48a**,**b**, however, confirming that arranging the binding sites in the substituents closer to the cyclopeptide allows the NH groups along the ring to contribute to anion binding. Remarkably, no obvious reduction of the sulfate affinity of **48b** was observed even when performing the binding studies in highly competitive water/DMSO mixtures up to 25 vol% of water. Unfortunately, the solubility of these cyclopeptides prevented binding studies in solvents with an even higher water content.

## 7. Cyclic Peptides Not Containing α-Amino Acid-Derived Subunits

**Figure 19:**
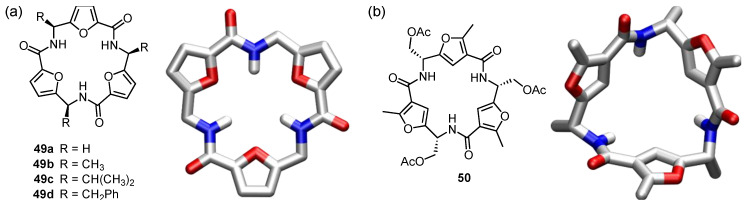
Structures and preferred calculated conformations of the furan-containing cyclopeptides **49a**–**d** (**a**) and **50** (**b**). The structures of **49a** and **50** were calculated by the author of this review based on the reported results by using Spartan 20 (Wavefunction, Inc., Irvine, CA, USA) and the MMFF force-field (see Supporting Information). The side chains of **50** are truncated and protons, except acidic ones, are omitted for reasons of clarity.

Cyclopeptide-based receptors have also been described featuring only abiotic amino acids along the ring. Somewhat related to the compounds described in the previous section are the furan-derived cyclopeptides **49a**–**d** described by Chakraborty and coworkers [175,176] and the structurally related analog **50** developed in the Robina group (Figure 19a) [177]. According to force-field calculations, **49a** preferentially adopts an almost planar *C*_3_ symmetric conformation with converging NH groups. The cyclopeptide is therefore preorganized for anion binding and binds acetate in acetonitrile with a log *K*_a_ of 3.9. The chiral analogs **49c**–**d** have similar conformations, although the side chains induce a tilted arrangement of the furan subunits, which thus surround a bowl-shaped cavity. With a log *K*_a_ of 3.7, the acetate affinity of **49d** in acetonitrile is slightly lower than that of **49a**. Chloride is bound with a log *K*_a_ of 3.9 under the same conditions. The cyclopeptides **49b**–**d** also exhibit antimicrobial activity against several bacterial strains [176].

Shifting the aminomethyl group from the 5-position in **49** to the 4-position in **50** also results in a tilted arrangement of the furan moieties (Figure 19b) [177]. The converging arrangement of the NH groups is retained, however, and **50** thus also interacts with anions. Chloride is bound with a log *K*_a_ of 4.2 in acetonitrile while the complexes with acetate (log *K*_a_ = 3.0) and cyanide (log *K*_a_ = 3.2) are less stable.

**Scheme 12:**
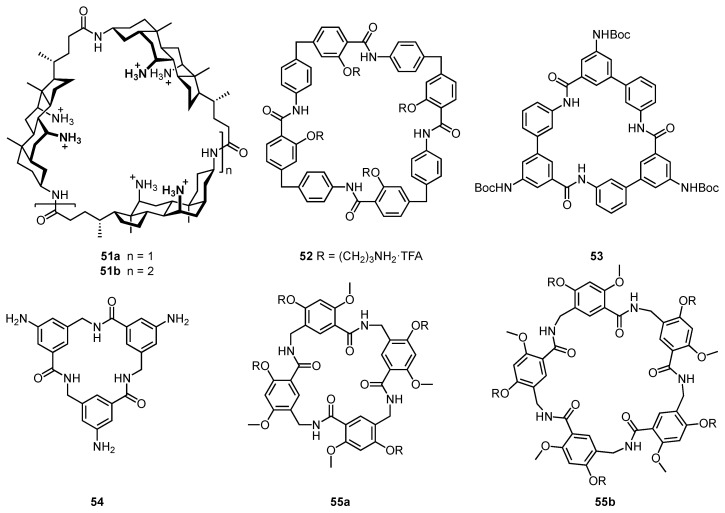
Structures of the cyclopeptides **51**–**55**.

Steroid-based amino acids were incorporated as rigid hydrophobic spacers into cyclopeptides for conformational control [178] and to use the inwardly directed functional groups in the axial positions of the steroidal framework as binding sites. The Davis group described in this context the so-called cyclocholamides **51a**,**b** (Scheme 12) [179].

Although both compounds are conformationally flexible due to the alkyl linkers in the subunits, they prefer to adopt toroidal conformations with the ammonium groups oriented toward the interior of the cavities. These compounds thus combine a hydrophobic exterior and a polar, positively charged interior, explaining their ability to transport chloride anions across lipid membranes, either through a carrier mechanism or by stacking within the bilayer to form pores. Independent work later suggested that related macrocycles derived from cholic acid in which hydroxy groups line the inner cavity have a tendency to stack within membranes and to thus produce pores [180,181].

**Figure 20:**
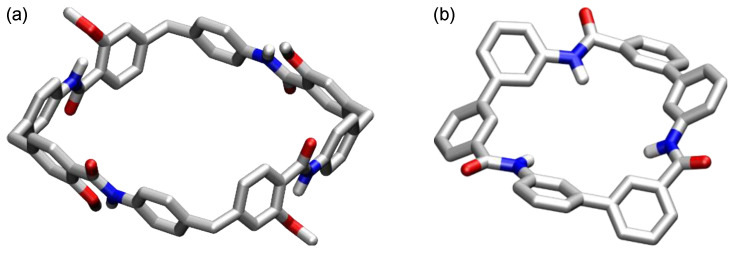
Calculated structures of cyclopeptides **52** (**a**) and **53** (**b**). The calculations were performed by the author of this review based on the reported results by using Spartan 20 (Wavefunction, Inc., Irvine, CA, USA) and the MMFF force-field (see Supporting Information). The side chains of all cyclopeptides are truncated and protons, except acidic ones, are omitted for reasons of clarity.

The cyclopeptides **52** and **53** (Scheme 12) solely contain aromatic building blocks. The cyclic tetramer **52**, which was described by Nowick and coworkers [182], contains four ι-amino acids (ι = iota). These subunits can be considered to represent analogs of glycine in which the *para*-substituted aromatic moieties serve to elongate the bonds from the central methylene unit to the flanking functional groups. The alkoxy groups stabilize conformations with intramolecular hydrogen bonds between the amide NH groups and the oxygen atoms, enhance solubility, and provide structural diversity. This cyclopeptide adopts a *C*_4_ symmetric conformation in DMSO-*d*_6_ with four *trans*-amides (Figure 20a). In D_2_O, two conformers exist in a ratio of circa 1:3, of which the all-*trans*-amide conformer is the minor one while the major conformer has a rectangular shape due to alternating *cis*-amide and *trans*-amide bonds. The complexation of sodium cholate in water shifts the conformational equilibrium toward the *C*_4_ symmetric conformation. Complex formation is associated with a log *K*_a_ of 4 and mediated by the hydrophobic effect and electrostatic interactions between the anionic substrate and the cationic host. The Nowick group also described a family of cyclopeptides containing biphenyl-derived amino acids [183], but the binding properties of these compounds were not evaluated.

The macrocyclic peptide **53** was developed by Hamilton and coworkers [184]. It features three NH groups pointing toward the center of the ca. 5 Å wide cavity (Figure 20b). This receptor binds tosylate in the form of a 1:1 complex in 2 vol% DMSO-*d*_6_/CDCl_3_ with a log *K*_a_ of 5.2. Inorganic anions such as halides, nitrate, hydrogensulfate, and dihydrogenphosphate induce the formation of 2:1 anion complexes, in which an anion is sandwiched between two receptor subunits like in the complexes of cyclopeptide **16a**. Increasing the solvent polarity reduces anion affinity significantly and simplifies the binding mode [185]. The log *K*_a_ of the tosylate affinity only amounts to 2.9 in DMSO-*d*_6_, for example, and halide and nitrate binding is almost completely lost. However, the affinity of **53** for tetrahedral anions is retained in the competitive medium, in which sulfate and dihydrogenphosphate complexation affords 1:1 complexes. Under these conditions, the sulfate complex has a log *K*_a_ of 3.2 while the log *K*_a_ of the dihydrogenphosphate amounts to 4.2. The anion selectivity of **53** was correlated with the size and shape of the receptor cavity. Accordingly, iodide binds more strongly to **53** in 2 vol% DMSO-*d*_6_/CDCl_3_ than chloride, despite the fact that the charge density of chloride is higher, because iodide better fits into the receptor cavity and can thus interact with all three NH groups simultaneously.

The Hamilton groups also developed the cyclopeptides **54** [186] and **55a**,**b** [187] (Scheme 12) of which the latter are particularly interesting because they can potentially adopt conformations due to intramolecular hydrogen bonds resembling the preferred ones of resorcin[4]arenes or pillar[5]arenes. Both compounds are flexible in solution, adopting *C*_4_ and *C*_5_ symmetrical conformations on average, but have nonsymmetric structures in the solid state. These cyclopeptides were used as scaffolds to arrange a sequence of side chains along the rings but have so far not been studied with respect to receptor properties.

## 8. Conclusions and Outlook

Natural cyclopeptides contain many subunits beyond the twenty proteinogenic amino acids that help stabilizing certain bioactive conformations, and chemists have almost unlimited possibilities to extend this structural diversity [135]. Possible strategies are the incorporation of abiotic building blocks into the cyclopeptide ring, the replacement of peptide groups with structural surrogates, or changing the directionality of the peptide bonds. While work in this area has largely focused, and still focuses, on identifying new lead structures for drugs or improving the properties of known bioactive compounds, cyclopeptides have also evolved into a potent class of synthetic receptors. Early work started even before the field of supramolecular chemistry was firmly established, but the basic concepts that were initially established remained valid for much of the work performed since then. Exerting control over the preferred cyclopeptide conformation by the careful choice of the building blocks is one of these concepts, and while it may not be always possible to reliably predict the outcome of a structural change, the tuning of receptor conformations when using cyclopeptide-based receptors allows for subtle control over binding properties [188], maybe an even more precise one than when using receptors with more restricted conformational freedom such as calixarenes or cyclodextrins. The sequential synthesis of cyclopeptides is also advantageous as it allows the relatively facile replacement of individual subunits.

With the progress made over the years, cyclopeptide-based receptors have become available that not only bind metal ions, thus allowing their use as synthetic ionophores, but that also recognize many other guests. Although the overall substrate scope is broad, charged guests likely play a larger role than neutral ones. In addition, a progressive shift of interest from cation to anion binders can be seen in recent years [8]. In addition to these developments, cyclopeptide-based systems have found other applications in supramolecular chemistry, some mentioned in the previous sections, such as the use in sensors, as building blocks of molecular motors, or as stimuli-responsive systems.

Today, cyclopeptide-based receptors are integral to supramolecular chemistry, but their potential is far from fully explored. Of the abiotic cyclopeptides already known, only a fraction has been investigated with respect to binding properties, for example, although many compounds have conformations that could qualify them as receptors. Not only can these systems give rise to new binders, but receptor development can also involve improving the properties of known systems or the invention of entirely new ones. Future work in this area can therefore take advantage of a wide range of possibilities.

## Data Availability

No new data were created or analyzed for this article, with the exception of the calculations performed to illustrate the structures shown in Figure 1, Figure 3, Figure 4, Figure 7, Figure 19 and Figure 20. The xyz files of the respective structures are available as Supporting Information.

## Supplementary Materials

The following supporting information can be downloaded at: https://www.mdpi.com/article/10.3390/molecules27092821/s1, xyz files of the structures shown in Figure 1, Figure 3, Figure 4, Figure 7, Figure 19 and Figure 20.
